# Tetraphenylpyrazine-based AIEgens: facile preparation and tunable light emission†
†Electronic supplementary information (ESI) available: Synthesis, characterization, TGA curves, PL spectra, and crystal structures of TPP and its derivatives. CCDC 1031716–1031719. For ESI and crystallographic data in CIF or other electronic format see DOI: 10.1039/c4sc03365e



**DOI:** 10.1039/c4sc03365e

**Published:** 2014-12-11

**Authors:** Ming Chen, Lingzhi Li, Han Nie, Jiaqi Tong, Lulin Yan, Bin Xu, Jing Zhi Sun, Wenjing Tian, Zujin Zhao, Anjun Qin, Ben Zhong Tang

**Affiliations:** a MOE Key Laboratory of Macromolecular Synthesis and Functionalization , Department of Polymer Science and Engineering , Zhejiang University , Hangzhou 310027 , China . Email: qinaj@zju.edu.cn; b Guangdong Innovative Research Team , State Key Laboratory of Luminescent Materials and Devices , South China University of Technology , Guangzhou 510640 , China; c Department of Chemistry , Institute for Advanced Study , Institute of Molecular Functional Materials, and State Key Laboratory of Molecular Neuroscience , The Hong Kong University of Science & Technology , Clear Water Bay , Kowloon , Hong Kong , China . Email: tangbenz@ust.hk; d State Key Laboratory of Supramolecular Structure and Materials , Jilin University , Changchun 130012 , China

## Abstract

Research on aggregation-induced emission (AIE) has been a hot topic. Due to enthusiastic efforts by many researchers, hundreds of AIE luminogens (AIEgens) have been generated which were mainly based on archetypal silole, tetraphenylethene, distyrylanthracene, triphenylethene, and tetraphenyl-1,4-butadiene, *etc.* To enlarge the family of AIEgens and to enrich their functions, new AIEgens are in high demand. In this work, we report a new kind of AIEgen based on tetraphenylpyrazine (TPP), which could be readily prepared under mild reaction conditions. Furthermore, we show that the TPP derivatives possess a good thermal stability and their emission could be fine-tuned by varying the substituents on their phenyl rings. It is anticipated that TPP derivatives could serve as a new type of widely utilized AIEgen, based on their facile preparation, good thermo-, photo- and chemostabilities, and efficient emission.

## Abstract 2

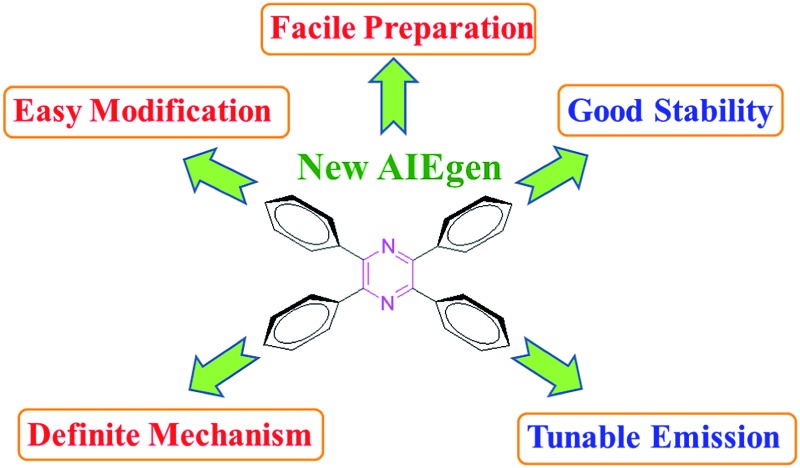
Structurally new AIEgens with tunable emission based on tetraphenylpyrazine were rationally designed and readily prepared.

## Introduction

Organic light-emitting materials are one of the key factors in the development of efficient organic light-emitting diodes, organic lasers, and fluorescent sensors, *etc.* However, the thorny problem encountered for the traditional organic light-emitting molecules is the well-known concentration/aggregation-caused quenching, which greatly limits the enhancement of device performance. One of the methods to surmount this difficulty is to let the aggregation play positive instead of negative roles in enhancing the efficiency in molecule emission in the condensed phases. Indeed, such a strategy has been successfully developed and is called aggregation-induced emission (AIE).^1^

Compared with traditional organic emitters, AIE luminogens (AIEgens) are a burgeoning class of conjugated molecules with propeller-shaped structures. They are weakly or non-luminescent in their dilute solutions, but emit efficiently when aggregated or fabricated into solid films.^2^ Due to their twisted conformation, the peripheral rotors, such as phenyl rings, of the AIEgens rotate against the central stator, such as (hetero)aromatic rings or vinyl groups, to annihilate the excitons in a non-radiative fashion in solution, while in the aggregate state this rotation is greatly restricted, which in turn opens up a radiative channel and makes the molecules emissive.^3^ This intriguing property enables AIEgens to be potentially applied in highly efficient optoelectronic devices, fluorescent sensors, cell imaging, and so on.

**Chart 1:**
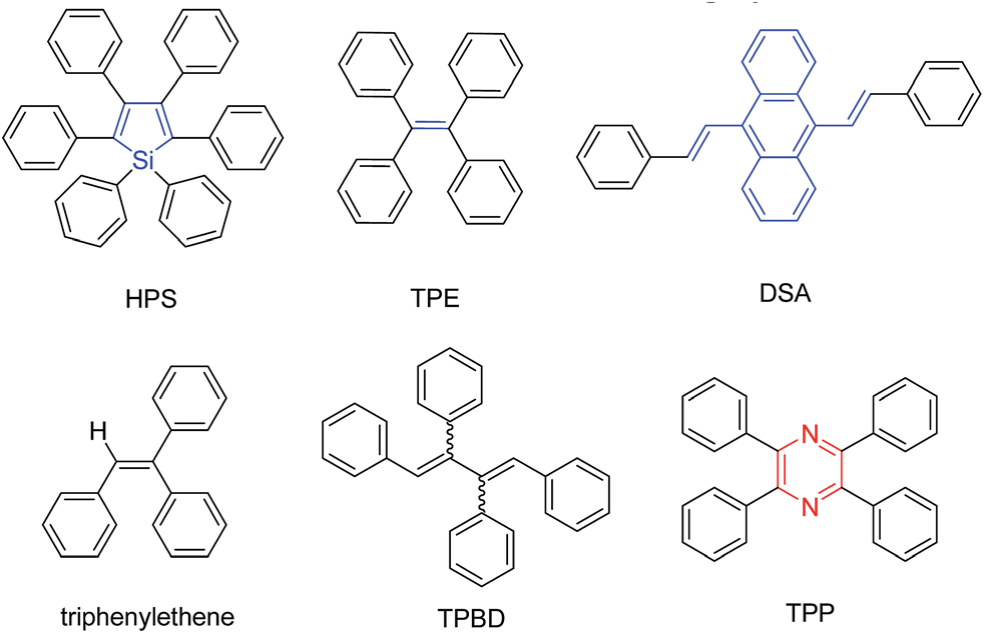
Molecular structures of AIEgens of hexaphenylsilole (HPS), tetraphenylethene (TPE), distyrylanthracene (DSA), triphenylethene, tetraphenyl-1,4-butadiene (TPBD) and tetraphenylpyrazine (TPP).

Thanks to enthusiastic efforts by many scientists, a lot of AIEgens with versatile functionalities and diverse applications have been developed (Chart 1).^4,5^ Currently, most AIEgens are derivatives of silole,^6^ tetraphenylethene (TPE),^7^ distyrylanthracene (DSA),^8^ triphenylethene,^9^ and tetraphenyl-1,4-butadiene (TPBD).^10^ Although these molecules could be regarded as the archetypal AIEgens, their intrinsic disadvantages should not be ignored. For example, silole and its derivatives are troublesome in preparation, especially in their purification, and unstable under basic conditions;^10*a*^ TPE, DSA, triphenylethene, TPBD and their derivatives contain double bonds, which may lead to potential photooxidation and photobleaching. The involved *E*/*Z* isomerization of the contained double bonds also complicates the mechanistic understanding, though we and others have theoretically and experimentally proven that the restriction of intramolecular rotation (RIR) is the cause for the AIE effect.^3^ Thus, new AIE cores, which are easy to synthesize and functionalize under mild reaction conditions and stable upon exposure to light and heat and under acidic and basic conditions, are highly desirable.^11^

In 2009, we developed a new AIEgen, 2,3-dicyano-5,6-diphenylpyrazine (DCDPP), which was synthesized under very mild reaction conditions in high yield by simply heating a mixture of dione and diamine in acetic acid.^12^ It is worth noting that there is no vinyl group present in DCDPP, which makes it more stable and greatly simplifies mechanism elucidation. Furthermore, the pyrazine ring is an electron-deficient group and could enrich the properties of the resultant AIEgens.^13^

Inspired by the facile preparation and encouraged by the AIE properties of DCDPP, in this work, we report a new type of AIEgen, tetraphenylpyrazine (TPP), which could be synthesized using a one-pot procedure under mild reaction conditions (its structure is shown in Chart 1). It is worth noting that the TPP core can be readily functionalized by varying the structure of the starting molecules or using post-reactions of the bromo-substituted TPP with aromatic boronic acid *via* Suzuki coupling, which provides an ideal platform to fine-tune the light emission of the resultant molecules from deep-blue to pure blue.

## Results and discussion

### Synthesis of TPP and its derivatives

**Scheme 1:**
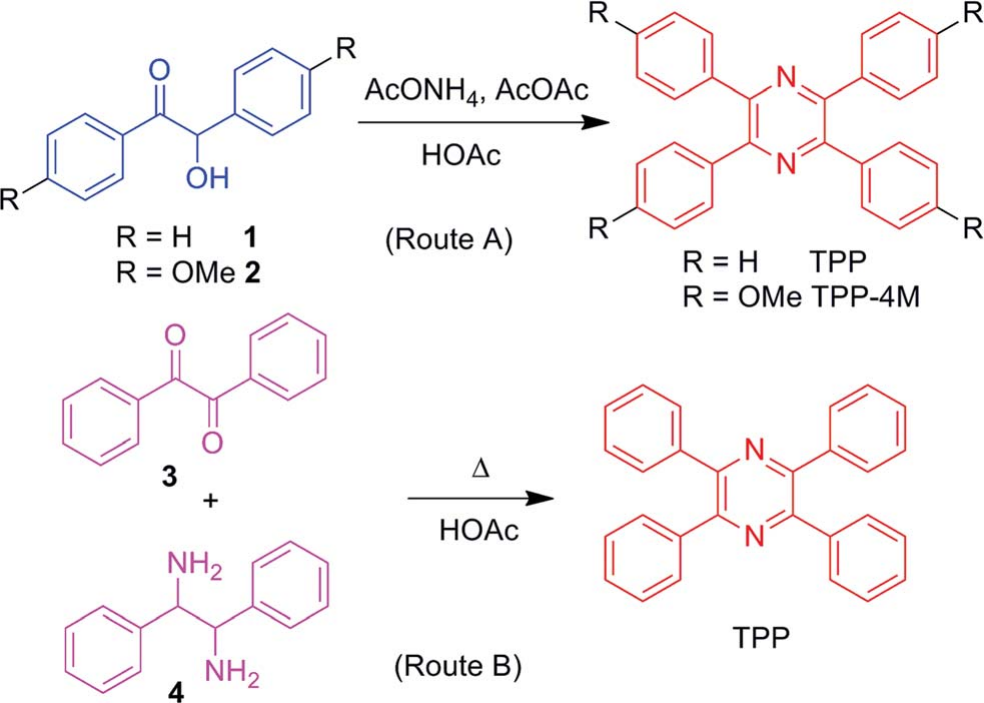
Synthetic routes to tetraphenylpyrazine (TPP) and its derivative.

In general, there are two routes to prepare TPP and its derivatives (Scheme 1). In route A, the commercially available starting compound benzoin (**1**) or anisoin (**2**) could be readily converted to TPP or its methoxy-substituted derivative, TPP-4M, in the presence of acetic anhydride and ammonium acetate after refluxing in acetic acid for only 3.5 h,^14^ whereas route B is similar to that in our previous report.^12,15^ Mixing benzil and 1,2-diphenylethane 1,2-diamine in acetic acid and refluxing the solution for 4 h produced TPP in moderate yield. More importantly, the produced TPP and its derivatives obtained *via* both routes could be purified using recrystallization and no tedious column chromatography was needed, making these routes the simplest for the preparation of AIEgens.

**Scheme 2:**
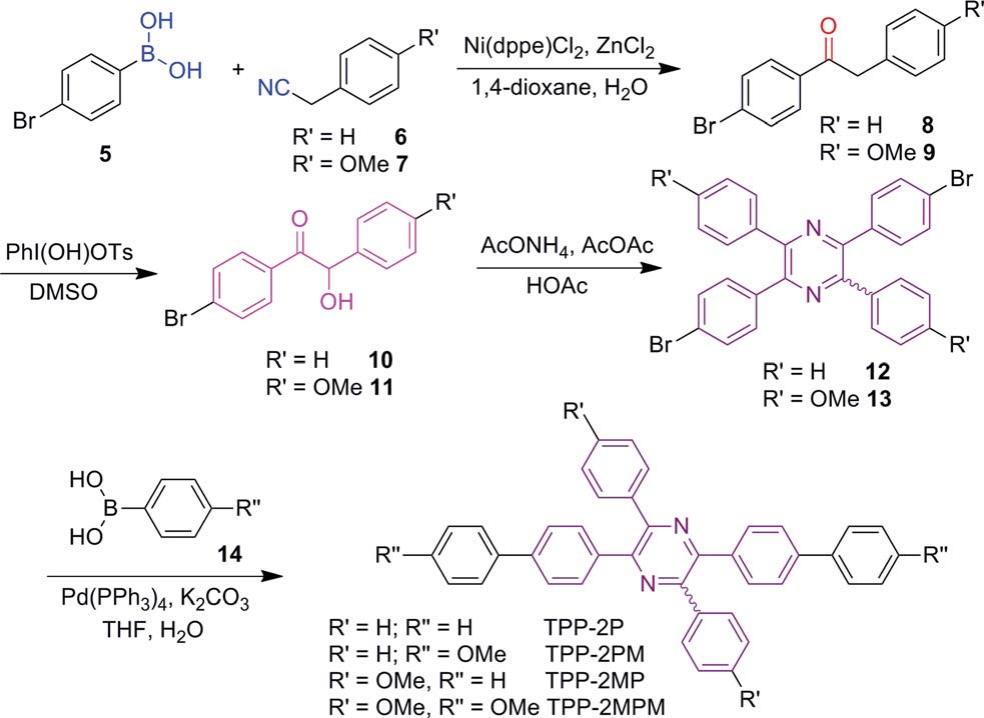
Synthetic routes to TPP-based AIEgens.

To verify the universality of these routes, we applied them to synthesize various TPP derivatives. In this paper, we will focus on route A and demonstrate its advantages (Scheme 2).Route B will be used in another work. The bromo-substituted intermediates **12** and **13**, shown in Scheme 2, could be readily prepared *via* three steps from commercially available bromo-substituted aromatic boronic acid and phenylacetonitrile using three successive catalytic systems of Ni/Zn, PhI(OH)OTs, and AcONH_4_/AcOAc. Afterwards, the Suzuki coupling reaction between the resultant intermediate and aromatic boronic acid **14** readily furnished the TPP derivatives with donor–π–acceptor structures in satisfactory yields. Detailed synthetic procedures are provided in the ESI.†

All TPP derivatives were characterized using ^1^H and ^13^C NMR spectra (Fig. S1–S24, ESI†) and high resolution mass spectra (Fig. S25–S30, ESI†). Satisfactory data corresponding to their structures were obtained. These TPP derivatives structurally exclude any unstable vinyl groups and are thermally stable. Taking TPP as an example, the temperature for 5% loss of its weight (*T*_d_) is 275 °C, which is quite similar to that of HPS and DSA, but is 62 °C higher than that of TPE (Fig. S31, ESI†). Interestingly, functionalization of TPP could further enhance *T*_d_ of the resultant products by 60 °C (Fig. S32, ESI†), which will increase the device stability if the compounds are applied in this field. It is worth noting that unlike HPS, TPP remains intact under both acidic and basic conditions as suggested by their remaining PL intensities in the aggregate states (Fig. S33, ESI†). Moreover, unlike the photo-oxidizable DSA (Fig. S34, ESI†), no new peak could be found in the ^1^H NMR spectrum of TPP after it was irradiated with a UV lamp at 365 nm with a power of 1.10 mW cm^–2^ for 2 h (Fig. S35, ESI†), demonstrating that it possesses good photostability.

### Absorption of TPP-based AIEgens

**Fig. 1:**
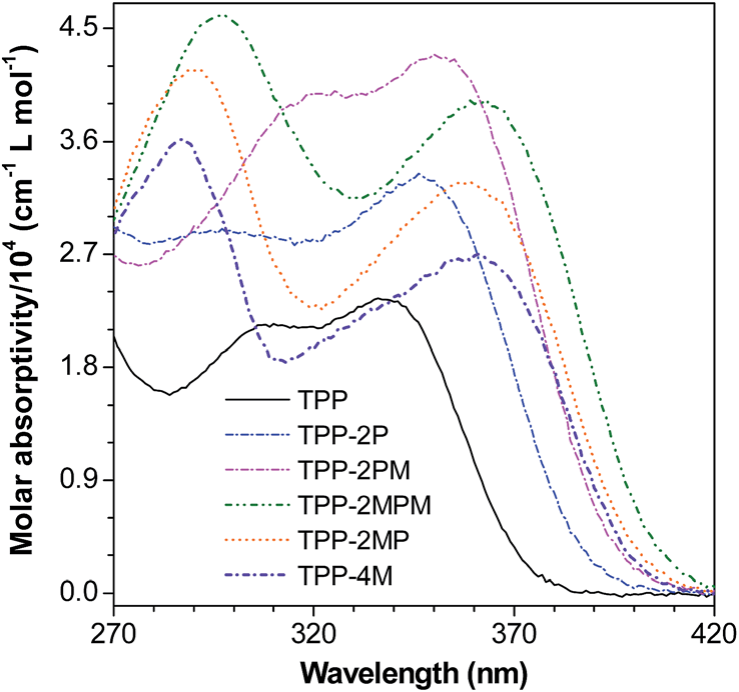
UV-vis absorption of TPP and its derivatives in THF.

After confirming the structures of TPP and its derivatives, we investigated their photophysical properties. It is well-known that pyrazine is an electron withdrawing group, thus, substitution of the phenyl rings of TPP with additional phenyl rings or electron-donating methoxyl groups will extend the conjugation and facilitate the charge transfer from the phenyl or methoxyl groups to the central pyrazine ring, and in turn red-shift the maximum absorption (*λ*_ab_) (Fig. 1 and Table S1, ESI†). Indeed, from TPP to TPP-2P, TPP-2PM and TPP-2MPM, enlarging the conjugated system and introducing D–A structures by incorporating phenyl and methoxyl groups have red-shifted *λ*_ab_ gradually from 338 to 362 nm. Meanwhile, when the methoxyphenyl groups were directly attached to the pyrazine ring (TPP-4M, TPP-2MP, and TPP-2MPM), longer *λ*_ab_ values with very little difference (2–3 nm) were observed, suggesting that the charge transfer plays a crucial role in controlling the absorption.

### Aggregation-enhanced emission

**Fig. 2:**
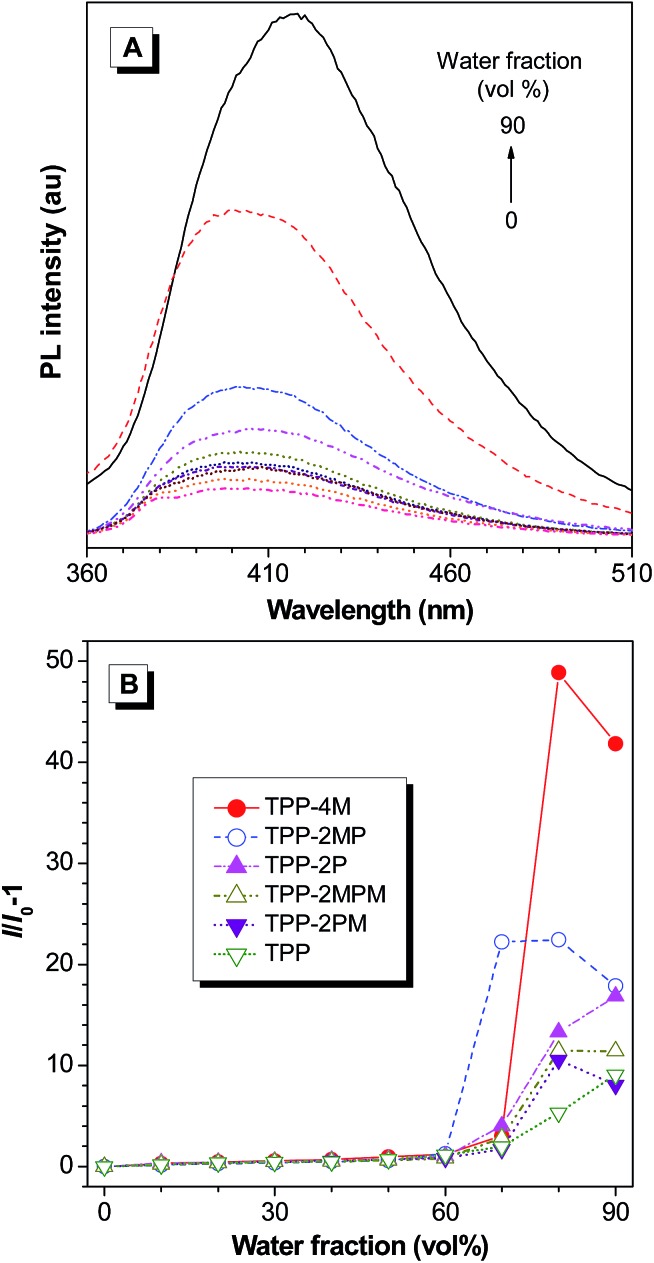
(A) PL spectra of TPP in THF–water mixtures with different water fractions; *λ*_ex_ = 338 nm (10 μM). (B) Variation in the PL intensity of TPP and its derivatives in THF–water mixtures with different water fractions.

By inspecting the structures of the resultant molecules, we can find that the phenyl rings are connected to the pyrazine core *via* single bonds. According to the RIR mechanism of AIE, these TPP derivatives could also feature the AIE effect. To confirm this, we studied their photoluminescence (PL) behaviours (Fig. 2 and S36–S40, ESI†). Fig. 2A shows the PL spectra of TPP in THF–water mixtures with different water fractions (*f*_w_) as an example. The emission of TPP is weak in THF solution and increases slowly until *f*_w_ reaches 60%. Afterwards, the emission intensified swiftly. Other TPP derivatives behave similarly: they are weakly emissive in THF solution and in THF–water mixtures with *f*_w_ lower than 60%. When *f*_w_ was beyond 60%, their emission enhanced remarkably. Since water is a poor solvent for TPP and its derivatives, the addition of water will induce the formation of nanoaggregates with sizes in the range of 31 to 287 nm (Table S2†). Thus, TPP and its derivatives feature the unique aggregation-enhanced emission (AEE) characteristics.

Moreover, when carefully inspecting Fig. 2B, we could observe that only the PL intensities of TPP and TPP-2P continue to increase in the THF–water mixtures with a *f*_w_ of 90%. The decrease in PL intensity of the other TPP-based AIEgens is related to the presence of methoxyl groups, which probably lead to the formation of less emissive amorphous nanoaggregates *via* random packing of the molecules in THF–water mixtures with 90% water fraction.^16^

It is worth noting that the emission enhancement of TPP-2P, TPP-2PM, TPP-2MPM and TPP-2MP is much higher than that of TPP (Fig. 2B) due to the presence of additional phenyl rings, which will reinforce the RIR. The absolute quantum yield (*Φ*_F_) measurements showed that these AIEgens possess high *Φ*_F_ values in the range of 8.3–30.7% and most of them are above 15% (Table S1, ESI†).

Interestingly, the highest emission enhancement was recorded for TPP-4M in a THF/water mixture with a *f*_w_ of 80%, which is 49-fold higher than that in THF solution. Accordingly, the highest absolute *Φ*_F_ (30.7%) was also recorded for TPP-4M. The reason might be related to the molecular packing as discussed below.

### Single crystal packing

To understand the emission behaviours of these AIEgens, we tried to grow their single crystals. Delightfully, single crystals of TPP (CCDC 1031716), TPP-2P (CCDC ; 1031717), TPP-2PM (CCDC ; 1031718), and TPP-4M (CCDC ; 1031719) suitable for X-ray diffraction analyses were obtained. The analysis showed that all these TPP-based AIEgens adopt non-planar conformations with twisted angles between the phenyl rotors and pyrazine stators in the range of 33 to 66° (Fig. S41–S44, ESI†).

**Fig. 3:**
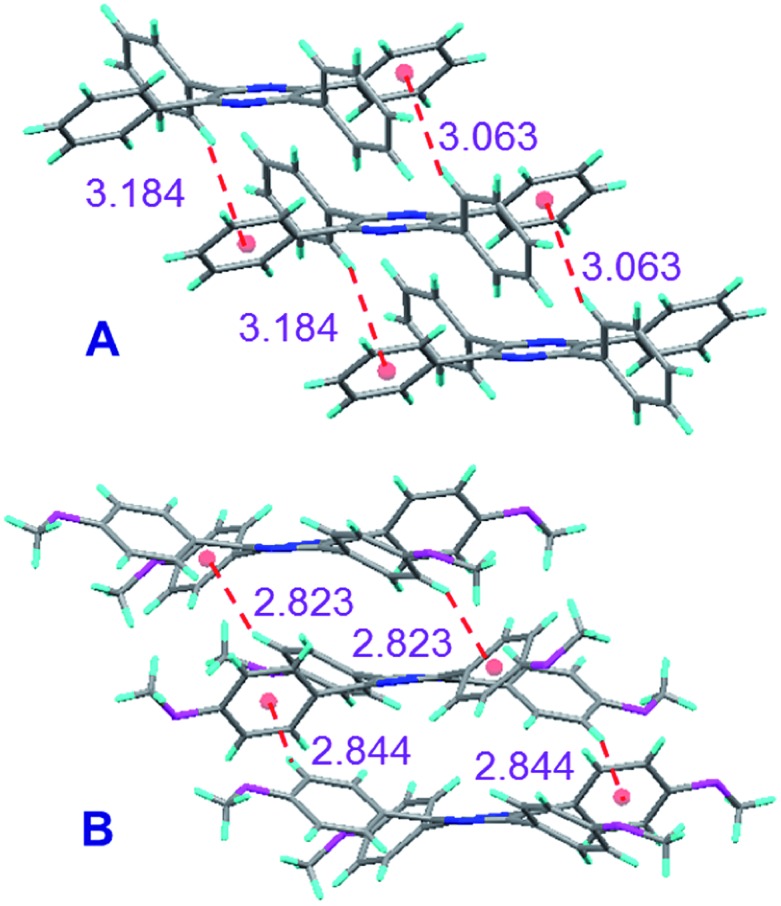
Single crystal packing diagrams and intermolecular C–H···π interactions of (A) TPP and (B) TPP-4M.

In a detailed study on the molecular packing in the single crystals, we could find that multiple intermolecular C–H···π interactions with distances in the range of 2.82–3.18 Å existed (Fig. 3). Such interactions could assist in locking the molecular motion in the crystal lattice and reducing the non-radiative deactivation of excitons.^12^ Moreover, the multiple C–H···π interactions in TPP-4M are much stronger than those in TPP because the interaction distances are much shorter in the former, which helped to enhance the molecular rigidity, limit the conformational freedom much more effectively and turn on its emission to a greater magnitude.

### Tunable blue emission

**Fig. 4:**
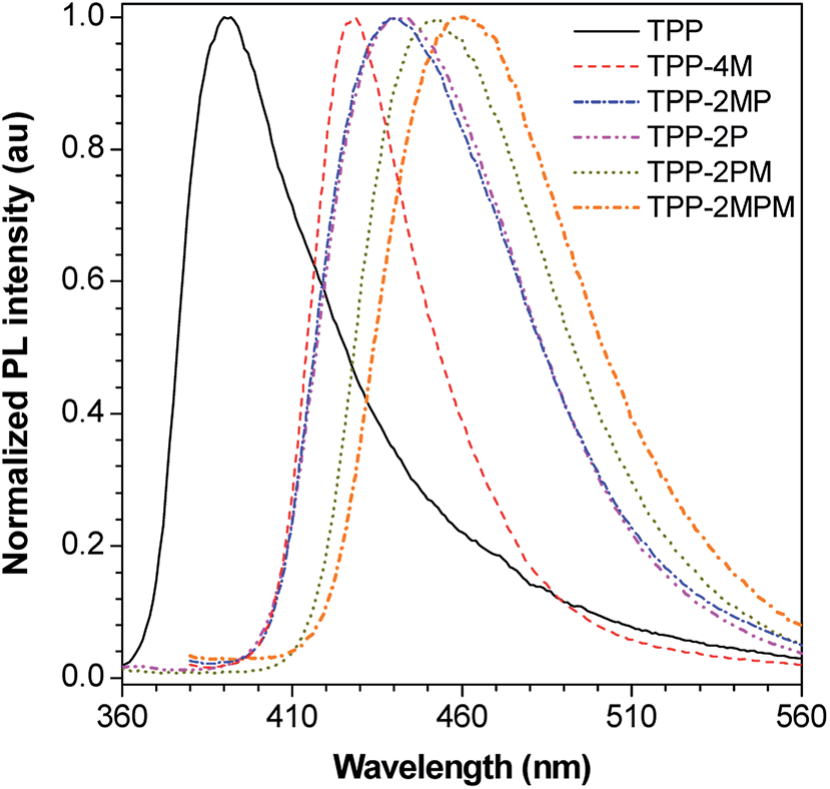
PL spectra of the thin films of TPP and its derivatives. The excitation wavelengths were selected based on the maximum absorption in THF, as shown in Table S1.†

Thanks to the facile functionalization, different TPP-based AIEgens emitting light from deep blue to pure blue could be obtained. As shown in Fig. 4 and S45 as well as Table S1 (ESI†), TPP in THF emits with a maximum peak at *ca.* 390 nm and TPP-2MPM at *ca.* 460 nm, whereas, in the thin-solid film, the emission peaks of these AIEgens remain almost unchanged due to the propeller-shaped molecular structures. Interestingly, from solution to film, the maximum emission peaks of TPP-2P and TPP-2PM red-shifted from 423 and 438 nm to 442 and 453 nm, whereas TPP-2MP and TPP-4M blue-shifted from 455 and 433 nm to 440 and 428 nm, respectively. The red-shift of emission peaks of TPP-2P and TPP-2PM implies that the number of additional phenyl rings connected to the TPP core plays a crucial role, which will probably cause additional intermolecular interactions going from solution to film. The blue-shift of the emissions of TPP-2MP and TPP-4M indicates that the charge-transfer from the methoxyl groups to the pyrazine ring was alleviated. Thus, these results suggest that the emission of TPP-based AIEgens could be fine-tuned by varying the substituents on TPP.

## Conclusions

In conclusion, a new type of AIEgen, TPP and its derivatives, was rationally designed according to the mechanism of RIR and the compounds were readily synthesized under mild reaction conditions. The TPP-based AIEgens simplify the mechanistic understanding because they do not contain any controversial vinyl groups. Furthermore, these AIEgens possess high thermo-, photo- and chemostabilities because they contain no active double bonds, as compared to TPE and DSA, and no instable Si–C bonds as compared to HPS under basic conditions. The emission of these AIEgens could be fine-tuned from deep-blue to pure blue by varying the substituents on the pyrazine rings. It is worth pointing out that the AEE-active TPP-based derivatives with electron accepting substituents, such as cyano-groups could also be readily prepared in high yield *via* method A (Scheme S1, Fig. S46 and S47), further manifesting the universality of this synthetic method. Moreover, thanks to the electron withdrawing ability of the pyrazine moiety, green or red emission is anticipated to be realized by attaching stronger electron donating groups onto the periphery of TPP. Thus, we believe that these TPP-based AIEgens will not only enrich the family of AIEgens and find wide application in light-emitting diodes, chemosensors and bioprobes *etc.*, but also attract considerable interest from scientists in the areas of fundamental photophysical research and materials science.
