# Supplementary material for: Tetraphenylpyrazine-based AIEgens: facile preparation and tunable light emission
Source: Chem Sci. 2014 Dec 11;6(3):1932–7. doi: 10.1039/c4sc03365e (PMC5501095; doi:10.1039/c4sc03365e)
Supplement: Supplementary file 1 [file SC-006-C4SC03365E-s001.pdf]

Electronic Supplementary Material (ESI) for Chemical Science.
This journal is © The Royal Society of Chemistry 2015

Electronic Supplementary Information

Tetraphenylpyrazine-Based AIEgens: Facile Preparation and Tunable Light Emission

Ming Chen,[a] Lingzhi Li,[a] Han Nie,[b] Jiaqi Tong,[a] Lulin Yan,[d] Bin Xu,[d] Jing Zhi Sun,[a] Wenjing Tian,[d] Zujin Zhao,[b] Anjun Qin,*[ab] and Ben Zhong Tang*[abc]

[a] MOE Key Laboratory of Macromolecular Synthesis and Functionalization, Department of Polymer Science and Engineering, Zhejiang University, Hangzhou 310027, China. E-mail: qinaj@zju.edu.cn

[b] Guangdong Innovative Research Team, State Key Laboratory of Luminescent Materials and Devices, South China University of Technology, Guangzhou 510640, China

[c] Department of Chemistry, Institute for Advanced Study, Institute of Molecular Functional Materials, and State Key Laboratory of Molecular Neuroscience, The Hong Kong University of Science & Technology, Clear Water Bay, Kowloon, Hong Kong China. E-mail: tangbenz@ust.uk

[d] State Key Laboratory of Supramolecular Structure and Materials, Jilin University, Changchun 130012, China.

Table of contents

Experimental Section S5

Figure S1. ^{1}H NMR spectrum of TPP in $CDCl_3$. The solvent peak is marked with asterisk. S10

Figure S2. ^{13}C NMR spectrum of TPP in $CDCl_3$. S11

Figure S3. ^{1}H NMR spectrum of TPP-4M in $CDCl_3$. The solvent peak is marked with asterisk. S11

Figure S4. ^{13}C NMR spectrum of TPP-4M in $CDCl_3$. The solvent peak is marked with asterisk. S12

Figure S5. ^{1}H NMR spectrum of **8** in DMSO-d_6. S12

Figure S6. ^{13}C NMR spectrum of **8** in $CDCl_3$. The solvent peak is marked with asterisk. S13

Figure S7. ^{1}H NMR spectrum of **9** in $CDCl_3$. The solvent peak is marked with asterisk. S13

Figure S8. ^{13}C NMR spectrum of **9** in $CDCl_3$. The solvent peak is marked with asterisk. S14

Figure S9. ^{1}H NMR spectrum of **10** in $CDCl_3$. The solvent peak is marked with asterisk. S14

Figure S10. ^{13}C NMR spectrum of **10** in $CDCl_3$. The solvent peak is marked with asterisk. S15

Figure S11. ^{1}H NMR spectrum of **11** in $CDCl_3$. The solvent peak is marked with asterisk. S15

Figure S12. ^{13}C NMR spectrum of **11** in $CDCl_3$. The solvent peak is marked with asterisk. S16

Figure S13. ^{1}H NMR spectrum of **12** in $CDCl_3$. The solvent peak is marked with asterisk. S16

Figure S14. ^{13}C NMR spectrum of **12** in $CDCl_3$. S17

Figure S15. ^{1}H NMR spectrum of **13** in $CDCl_3$. The solvent peak is marked with

asterisk. S17

Figure S16. ^{13}C NMR spectrum of **13** in $CDCl_3$. The solvent peak is marked with asterisk. S18

Figure S17. ^{1}H NMR spectrum of TPP-2P in $CDCl_3$. The solvent peak is marked with asterisk. S18

Figure S18. ^{13}C NMR spectrum of TPP-2P in $CDCl_3$. S19

Figure S19. ^{1}H NMR spectrum of TPP-2PM in $CDCl_3$. The solvent peak is marked with asterisk. S19

Figure S20. ^{13}C NMR spectrum of TPP-2PM in $CDCl_3$. The solvent peaks are marked with asterisk.

S20

Figure S21. ^{1}H NMR spectrum of TPP-2MP in $CDCl_3$. The solvent peak is marked with asterisk. S20

Figure S22. ^{13}C NMR spectrum of TPP-2MP in $CDCl_3$. The solvent peak is marked with asterisk. S21

Figure S23. ^{1}H NMR spectrum of TPP-2MPM in $CDCl_3$. The solvent peak is marked with asterisk. S21

Figure S24. ^{13}C NMR spectrum of TPP-2MPM in $CDCl_3$. The solvent peak is marked with asterisk. S22

Figure S25. HRMS spectra of TPP. S22

Figure S26. HRMS spectra of TPP-4M. S23

Figure S27. HRMS spectra of TPP-2P.

S23

Figure S28. HRMS spectra of TPP-2PM. S24

Figure S29. HRMS spectra of TPP-2MP. S24

Figure S30. HRMS spectra of TPP-2MPM.

S25

Figure S31. TGA curves of AIEgens under nitrogen at a heating rate of 10 ^{o}C/min.

S25

Figure S32. TGA curves of TPP and its derivatives under nitrogen at a heating rate of 10 °C/min. S26

Figure S33. PL spectra of pristine and treated TPP in THF/water mixtures with ~90% water fractions. Concentration: 10^{-5} M. λ_{ex}: 338 nm. The acid and base are 0.1 mL HCl or NaOH aqueous solution (1M), respectively. S26

Figure S34. ^{1}H NMR spectra of DSA before (A) and after (B) irradiation by a UV light of 365 nm for 2 h in $CDCl_3$. S27

Figure S35. ^{1}H NMR spectra of TPP before (A) and after (B) irradiation by a UV light of 365 nm with a power of 1.10 mW/cm^2 for 2 h in $CDCl_3$. The solvent peaks are marked with asterisk. S27

Figure S36. PL spectra of TPP-2P in THF/water mixtures with different water fraction. Concentration: 10^{-5} M, λ_{ex} = 347 nm. S28

Figure S37. PL spectra of TPP-2PM in THF/water mixtures with different water fraction. Concentration: 10^{-5} M, λ_{ex} = 352 nm. S28

Figure S38. PL spectra of TPP-2MPM in THF/water mixtures with different water fraction. Concentration: 10^{-5} M, λ_{ex} = 362 nm. S29

Figure S39. PL spectra of TPP-2MP in THF/water mixtures with different water fraction. Concentration: 10^{-5} M, λ_{ex} = 359 nm. S29

Figure S40. PL spectra of TPP-4M in THF/water mixtures with different water fraction. 10^{-5} M, λ_{ex} = 360 nm. S30

Figure S41. Molecular structures and torsion angles of TPP. S30

Figure S42. Molecular structures and torsion angles of TPP-4M. S31

Figure S43. Molecular structures and torsion angles of TPP-2P. S31

Figure S44. Molecular structures and torsion angles of TPP-2PM. S31

Figure S45. Normalized PL spectra of TPP derivatives in THF. Concentration: 10 μM. S32

Scheme S1. Synthetic route to TPP-2PCN. S32

Figure S46. ^{1}H NMR spectrum of TPP-2PCN in $CDCl_3$. S33

Figure S47. PL spectra of TPP-2PCN in THF/water mixtures with different water fraction. Concentration: 10^{-5} M, λ_{ex} = 347 nm. S33

Table S1. Optical and thermal properties of TPP and its derivatives. S34
Table S2. Particle sizes of TPP-based AIEgens in THF/water mixtures. S34
References S34

Experimental Section

Materials: All commercially available chemicals were purchased from Alfa Aesar, J&K chemistry or Sinopharm Chemical Reagent Co., Ltd and used directly without further purification. Tetrahydrofuran (THF) and 1,4-dioxane were distilled from sodium benzopheone ketyl under dry nitrogen immediately before use.

Instrumentation: All ^{1}H and ^{13}C NMR spectra were recorded with a Bruker AVANCE III 500 spectrometer using $CDCl_3$ or DMSO-d_6 as solvent. High resolution mass spectra (HRMS) were tested using a GCT premier CAB048 mass spectrometer operated in MALDI-TOF mode. UV-visible absorption spectra were measured with a Varian CARY 100 Biospectrophotometer. PL spectra were recorded on a RF-5301 PC spectrofluorometer. PL quantum efficiency (Φ_F) was measured with standard quinine sulfate in 0.1N H_2SO_4 ($\Phi_F = 0.54$) or anthracene in ethanol ($\Phi_F = 0.27$). The absolute Φ_F values were recorded with a Hamamatsu Quantaurus-QY C11347 spectrometer. Thermogravimetric analysis (TGA) was carried out with a PerkinElmer TGA 7 at a heating rate of 10 $^{o}C/min$ under dry nitrogen. Single crystal X-ray diffraction was carried out on a Gemini A Ultra diffractometer at 293K.

Synthesis of tetraphenylpyrazine (TPP): Rout A: The compound was synthesized accord to the literature with slight modification.[1] Into a 50 mL round bottom flask was added 2.12 g (10 mmol) of benzoin, 1.45 mL (15 mmol) of acetic anhydride, 2.32 g

(30 mmol) of ammonium acetate and 10 mL acetic acid. After refluxing for 3.5 h, the mixture was cooled down to room temperature and then filtered.

Route B[2]: Into a 50 mL round bottom flask was added 212 mg (1 mmol) of 1,2-diphenylethane-1,2-diamine, 210 mg (1 mmol) of benzyl and 2 mL of acetic acid. The mixture was allowed to reflux for 4 h. Afterwards, the mixture was cooled down to room temperature and filtered.

The crude products were purified with recrystallization in acetic acid three times. White crystal, yield: 33.9% (route A); 46.9% (route B). ^{1}H NMR (500 MHz $CDCl_3$): δ (TMS, ppm) 7.65 (m, 8H), 7.33 (m, 12H). ^{13}C NMR (125 MHz, $CDCl_3$): δ (TMS, ppm) 148.5, 138.5, 129.9, 128.7, 128.3. HRMS (MALDI-TOF): *m/z* 384.1664 ([M]$^+$), calcd for $C_{28}H_{20}N_2$ 384.1626).

Synthesis of TPP derivative (TPP-4M): The synthetic method was similar to that of TPP. White crystal, yield: 20 %. ^{1}H NMR (500 MHz $CDCl_3$): δ (TMS, ppm) 7.61 (d, 8H), 6.86 (d, 8H), 3.82 (s, 12H). ^{13}C NMR (125 MHz, $CDCl_3$): δ (TMS, ppm) 159.9, 146.8, 131.1, 113.7, 55.3. HRMS (MALDI-TOF): *m/z* 504.2039 ([M]$^+$), calcd for $C_{32}H_{28}N_2O_4$: 504.2049).

Synthesis of 1-(4-bromophenyl)-2-phenylethanone (8): The product was prepared according to the literature procedure.[3] Into a 250 mL round bottom flask was added 10 g (50 mmol) of 4-bromophenyl boronic acid (**5**), 1.32 g (2.5 mmol) of Ni(dppe)Cl_2 and 5.1 g (37.5 mmol) of $ZnCl_2$ under nitrogen. Then, 75 mL of 1,4-dioxane, 2.9 mL (25 mmol) of phenylacetonitrile (**6**) and 0.45 mL (25 mmol) of water was injected into the flask and the reaction was allowed to stir at 80 ^{o}C for 8 h. Afterward, the

mixture was filtered and the residue was washed with THF three times. Then the filtrate was concentrated and further dissolved in DCM, and washed with water to remove residual 1,4-dioxane. The collected organic phase was condensed and purified by a silica-gel column with ethyl acetate/hexane (1:20 by volume) as eluent. White solid 8 was obtained in 74.4% yield. ^{1}H NMR (500 MHz, DMSO-d$_6$): δ (TMS, ppm) 7.99 (d, 2H), 7.76 (d, 2H), 7.26 (m, 5H), 4.39 (s, 2H). ^{13}C NMR (125 MHz, $CDCl_3$): δ (TMS, ppm) 196.8, 135.5, 134.4, 132.2, 130.4, 129.6, 129.0, 128.6, 127.3, 45.8.

Synthesis of 1-(4-bromophenyl)-2-(4-methoxyphenyl)ethanone (9): The synthetic method was similar to that of **8**. White solid, yield: 67.0 %. ^{1}H NMR (500 MHz, $CDCl_3$): δ (TMS, ppm) 7.87 (d, 2H), 7.60 (d, 2H), 7.17 (d, 2H), 6.87 (d, 2H), 4.19 (s, 2H), 3.78 (s, 3H). ^{13}C NMR (125 MHz, $CDCl_3$): δ (TMS, ppm) 196.9, 158.6, 135.1, 131.9, 130.4, 130.2, 128.3, 126.1, 114.2, 55.3, 44.7.

Synthesis of 1-(4-bromophenyl)-2-hydroxy-2-phenylethanone (10): The product was prepared according to the literature procedure.[3] Into a 250mL round bottom flask was added 6 g (22 mmol) of **9**, 150 mL of dimethyl sulfoxide and 7.5 mL of water. After complete dissolution, 20.5 g (52.8mmol) of PHI(OH)OTs was added into the mixtures in two times. The reaction was kept at room temperature for 24 h. After reaction, the mixture was poured into the water and extracted with ethyl acetate. The collected organic phase was first washed with a large amount of water, and then condensed and purified by a silica-gel column with ethyl acetate/hexane (1:10 by volume) as eluent. White solid **10** was obtained in 35.4% yield. ^{1}H NMR (500 MHz, $CDCl_3$): δ (TMS, ppm) 7.78 (d, 2H), 7.54 (d, 2H), 7.32 (m, 5H), 5.89 (s, 1H) 4.47 (s,

1H). ^{13}C NMR (125 MHz, $CDCl_3$): δ (TMS, ppm) 198.3, 138.9, 132.3, 130.8, 129.5, 129.0, 128.0, 76.6.

Synthesis of 1-(4-bromophenyl)-2-hydroxy-2-(4-methoxyphenyl)ethanone (11): The synthetic method was similar to that of **10**. Yellow viscous liquid, yield: 34.4 %. ^{1}H NMR (500 MHz, $CDCl_3$): δ (TMS, ppm) 7.77 (d, 2H), 7.54 (d, 2H), 7.23 (d, 2H), 6.86 (d, 2H), 5.85 (s, 1H), 4.41 (s, 1H), 3.76 (s, 3H). ^{13}C NMR (125 MHz, $CDCl_3$): δ (TMS, ppm) 198.1, 159.9, 132.3, 132.0, 130.8, 130.6, 129.1, 114.7, 75.8, 55.3.

Synthesis of 12: The synthetic method was similar to that of TPP with method A. White solid, yield: 18.6 %. ^{1}H NMR (500 MHz, $CDCl_3$): δ (TMS, ppm) 7.61 (m, 4H), 7.50 (m, 4H), 7.46 (m, 4H), 7.36 (m, 6H). ^{13}C NMR (125 MHz, $CDCl_3$): δ (TMS, ppm) 148.6, 148.4, 147.4, 147.2, 138.0, 137.2, 131.5, 131.4, 129.8, 129.0, 128.5, 123.3.

Synthesis of 13: The synthetic method was similar to that of TPP with method A. White solid, yield: 23.5 %. ^{1}H NMR (500 MHz, $CDCl_3$): δ (TMS, ppm) 7.56 (m, 8H), 7.45 (d, 4H), 6.87 (d, 4H), 3.83 (s, 6H). ^{13}C NMR (125 MHz, $CDCl_3$): δ (TMS, ppm) 160.3, 148.0, 147.5, 146.8, 146.1, 137.6, 131.5, 131.4, 131.3, 131.2, 131.1, 130.3, 123.0, 113.9, 55.3.

Synthesis of TPP-2P: Into a 250 mL round bottom flask was added 120 mg (0.22 mmol) of **12**, 66 mg (0.54 mmol) of benzoboric acid, 12.8 mg (0.011 mmol) of $Pd(PPh_3)_4$, 10 mL of THF and 1.38 g of K_2CO_3 (dissolved in 5 mL of water) under nitrogen. The mixture was allowed for stir at 80 ^{o}C for12 h. After cooled down to room temperature, the mixture was diluted with dichloromethane and washed with

NH_4Cl aqueous solution. The collected organic phase was condensed and purified by a silica-gel column with ethyl acetate/hexane (1:50 by volume) as eluent. White solid, yield: 58.9 %. ^1H NMR (500 MHz, $CDCl_3$): δ(TMS, ppm) 7.74 (m, 8H), 7.64 (d, 4H), 7.59 (d, 4H), 7.45 (t, 4H), 7.37 (m, 8H). ^{13}C NMR (125 MHz, $CDCl_3$): δ(TMS, ppm) 148.6, 148.5, 148.2, 148.1, 141.5, 140.7, 138.7, 137.6, 130.6, 130.2, 129.1, 129.0, 128.6, 127.8, 127.3, 127.2. HRMS (MALDI-TOF): *m/z* 536.2248 ($[M]^+$), calcd for $C_{40}H_{28}N_2$ 536.2252).

Synthesis of TPP-2PM: The synthetic method was similar to that of TPP-2P. White solid, yield: 68.2 %. ^1H NMR (500 MHz, $CDCl_3$): δ(TMS, ppm) 7.72 (m, 8H), 7.57 (d, 4H), 7.52 (d, 4H), 7.35 (m, 6H), 6.97 (d, 4H), 3.85 (s, 6H). ^{13}C NMR (125 MHz, $CDCl_3$): δ(TMS, ppm) 159.6, 148.5, 148.4, 148.2, 148.0, 141.2, 138.8, 137.0, 133.2, 130.5, 130.1, 128.9, 128.6, 128.3, 126.7, 114.5, 55.6. HRMS (MALDI-TOF): *m/z* 596.2462 ($[M]^+$), calcd for $C_{42}H_{32}N_2O_2$ 596.2464).

Synthesis of TPP-2MP: The synthetic method was similar to that of TPP-2P. White solid, yield: 79.3 %. ^1H NMR (500 MHz, $CDCl_3$): δ(TMS, ppm) 7.77 (d, 4H), 7.66 (m, 8H), 7.60 (d, 4H), 7.45 (t, 4H), 7.36 (t, 2H), 6.89 (d, 4H), 3.83 (6H). ^{13}C NMR (125 MHz, $CDCl_3$): δ(TMS, ppm) 160.1, 147.7, 147.4, 147.2, 146.9, 141.1, 140.6, 137.9, 131.3, 130.9, 130.2, 128.8, 127.5, 127.1, 127.0, 113.8, 55.3. HRMS (MALDI-TOF): *m/z* 596.2457 ($[M]^+$, calcd for $C_{42}H_{32}N_2O_2$ 596.2464).

Synthesis of TPP-2MPM: The synthetic method was similar to that of TPP-2P. White solid, yield: 62.9 %. ^1H NMR (500 MHz, $CDCl_3$): δ(TMS, ppm) 7.74 (d, 4H), 7.65 (d, 4H), 7.58 (d, 4H), 7.53 (d, 4H), 6.99 (d, 4H), 6.88 (d, 4H), 3.86 (s, 6H), 3.83

(s, 6H). ^{13}C NMR (125 MHz, $CDCl_3$): δ (TMS, ppm) 160.1, 159.4, 147.5, 147.3, 147.2, 146.9, 140.6, 137.2, 133.0, 131.3, 131.0, 130.2, 128.1, 126.4, 114.3, 113.7, 55.4, 55.3. HRMS (MALDI-TOF): *m/z* 656.2658 ($[M]^+$, calcd for $C_{44}H_{36}N_2O_4$ 656.2675).

Synthesis of TPP-2PCN: Into a 100 mL round bottom flask was added 60 mg (0.11 mmol) of **12**, 48 mg (0.33 mmol) of **15** and 12.8 mg (0.011 mmol) of $Pd(PPh_3)_4$, 10 mL of toluene, 5 mL of EtOH and 1.38 g K_2CO_3 (dissolved in 5 mL of water) under nitrogen. The mixture was reacted at 110 ^{o}C for 6 h. Afterwards, solvent was removed by reduced pressure distillation and the residue was washed with dichloromethane and water. The collected organic phases were concentrated and the product was purified by a silica-gel column with dichloromethane/hexane (4:5 by volume) as eluent. White solid TPP-2PCN was obtained in 80% yield. ^{1}H NMR (300 MHz, $CDCl_3$): δ (TMS, ppm) 7.72 (m, 16H), 7.59 (m, 4H), 7.39 (m, 6H).

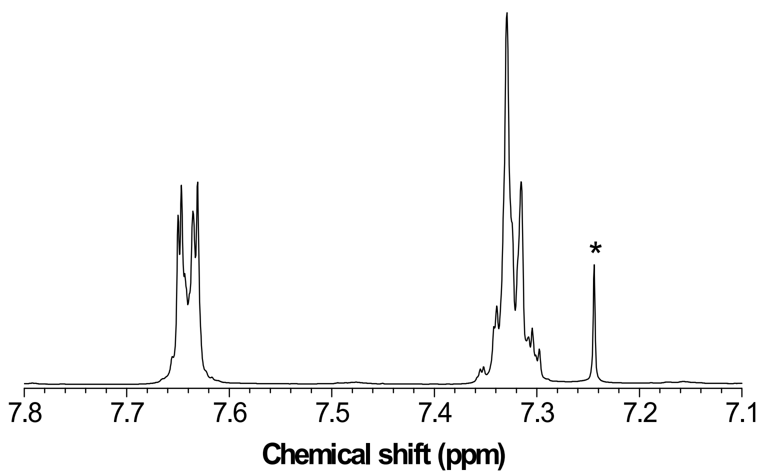

Figure S1. ^{1}H NMR spectrum of TPP in $CDCl_3$. The solvent peak is marked with asterisk.

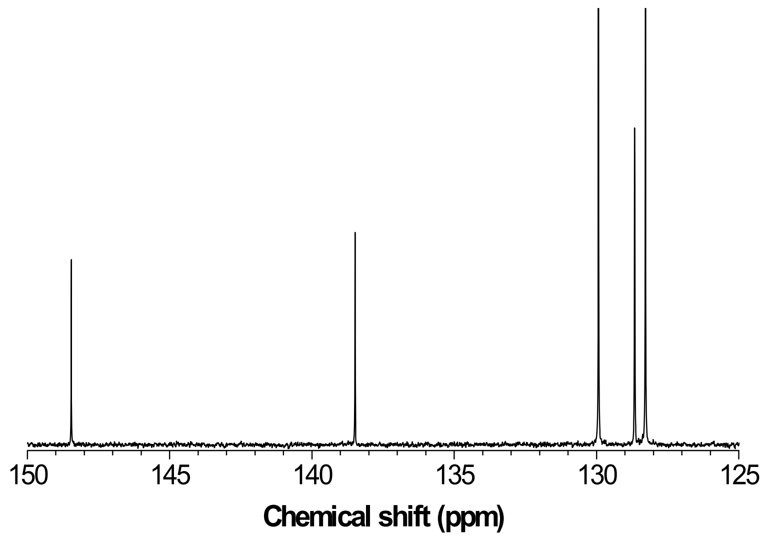

Figure S2. ^{13}C NMR spectrum of TPP in $CDCl_3$.

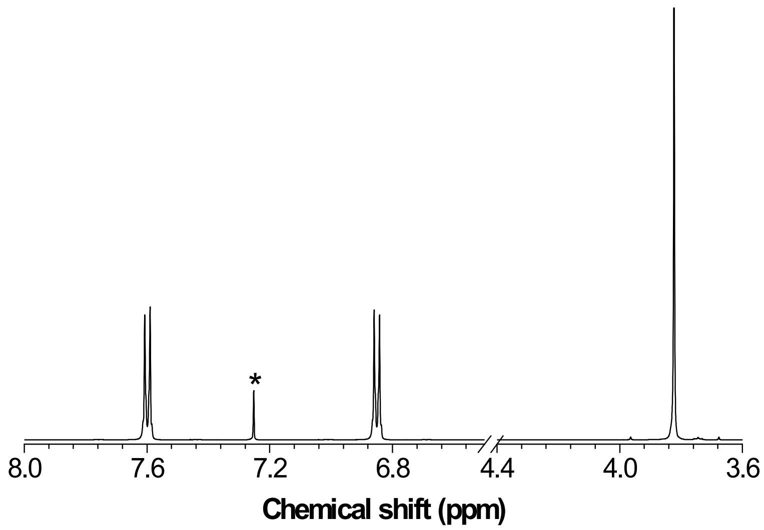

Figure S3. ^{1}H NMR spectrum of TPP-4M in $CDCl_3$. The solvent peak is marked with asterisk.

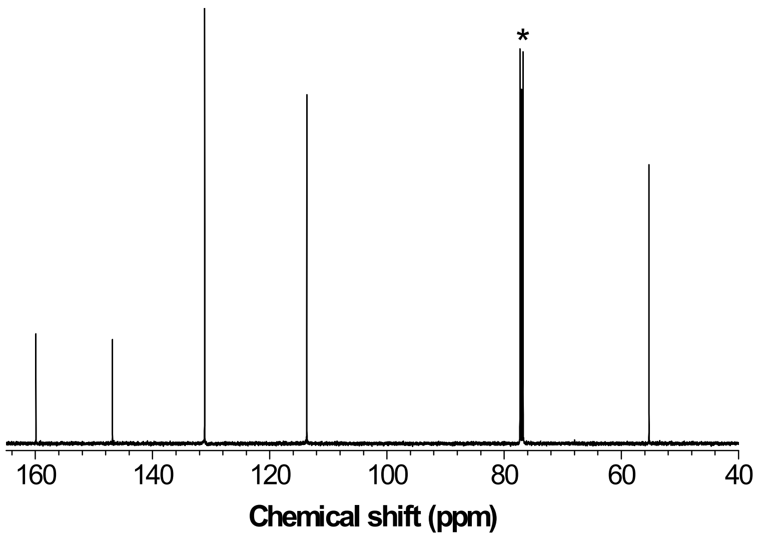

Figure S4. ^{13}C NMR spectrum of TPP-4M in $CDCl_3$. The solvent peak is marked with asterisk.

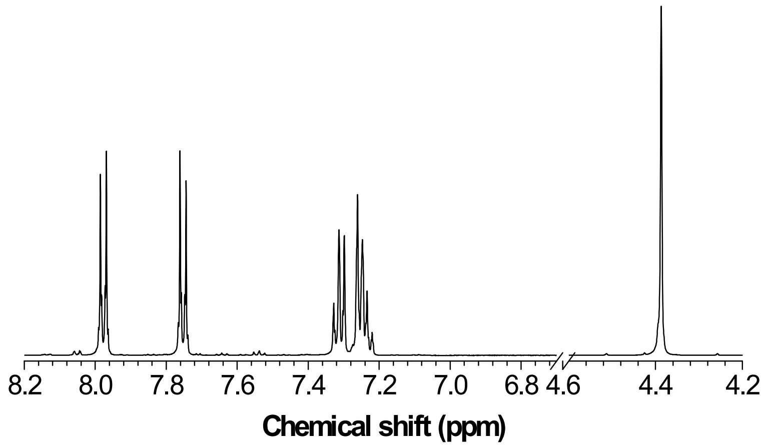

Figure S5. ^{1}H NMR spectrum of **8** in DMSO-d_6.

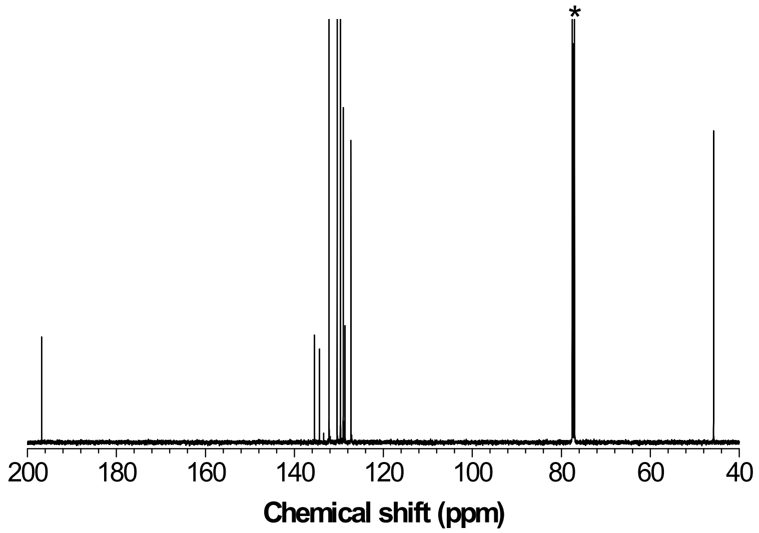

Figure S6. ^{13}C NMR spectrum of **8** in $CDCl_3$. The solvent peak is marked with asterisk.

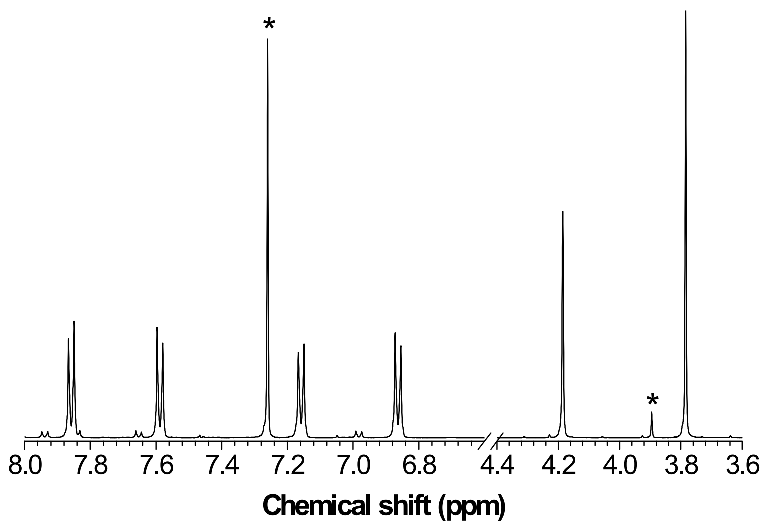

Figure S7. ^{1}H NMR spectrum of **9** in $CDCl_3$. The solvent peak is marked with asterisk.

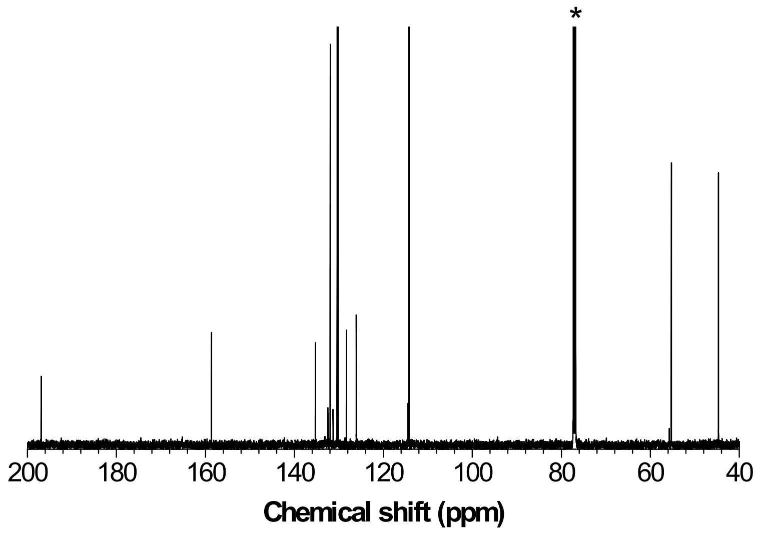

Figure S8. ^{13}C NMR spectrum of **9** in $CDCl_3$. The solvent peak is marked with asterisk.

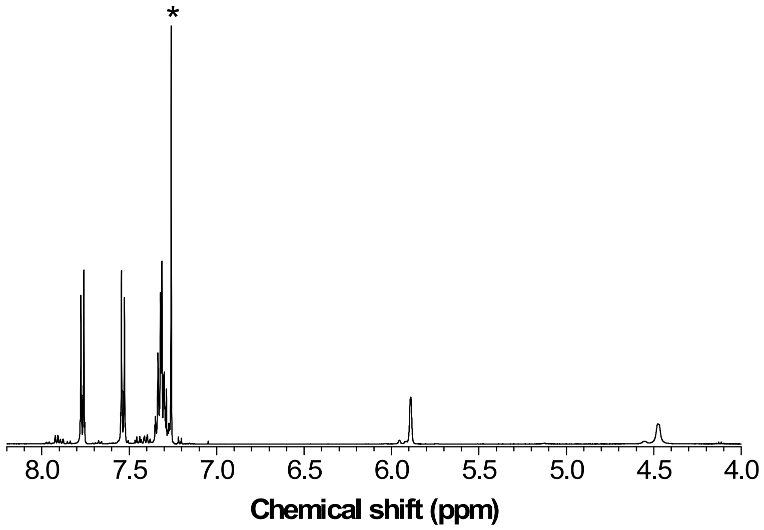

Figure S9. ^{1}H NMR spectrum of **10** in $CDCl_3$. The solvent peak is marked with asterisk.

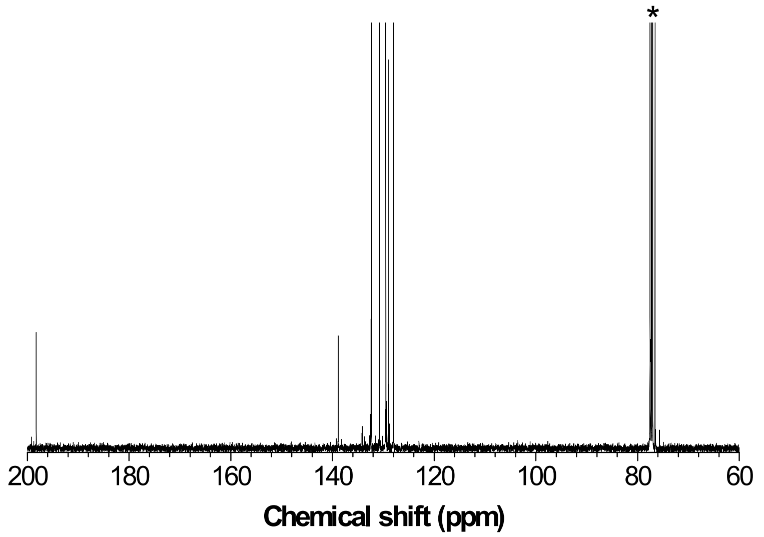

Figure S10. ^{13}C NMR spectrum of **10** in $CDCl_3$. The solvent peak is marked with asterisk.

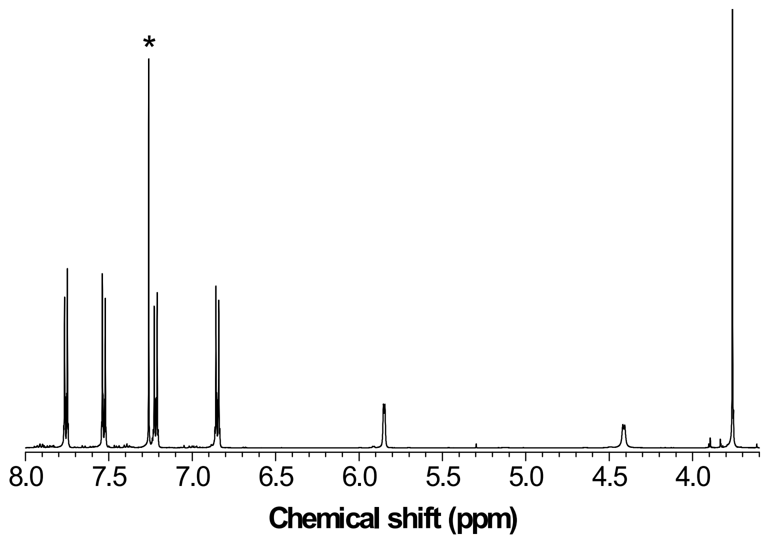

Figure S11. ^{1}H NMR spectrum of **11** in $CDCl_3$. The solvent peak is marked with asterisk.

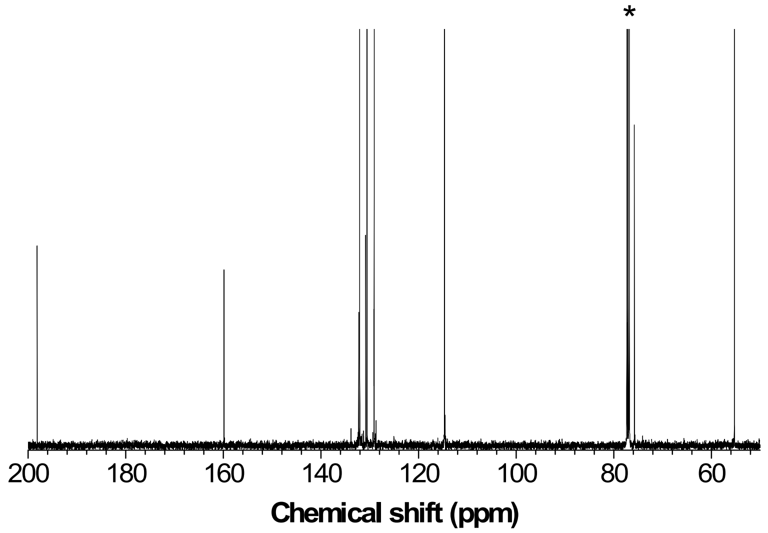

Figure S12. ^{13}C NMR spectrum of **11** in $CDCl_3$. The solvent peak is marked with asterisk.

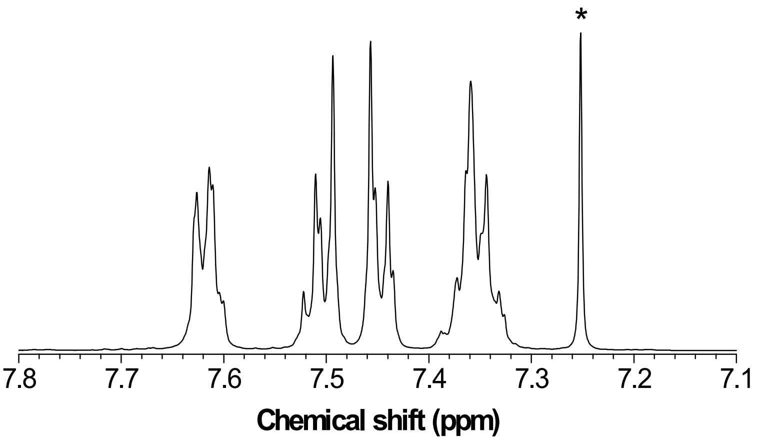

Figure S13. ^{1}H NMR spectrum of **12** in $CDCl_3$. The solvent peak is marked with asterisk.

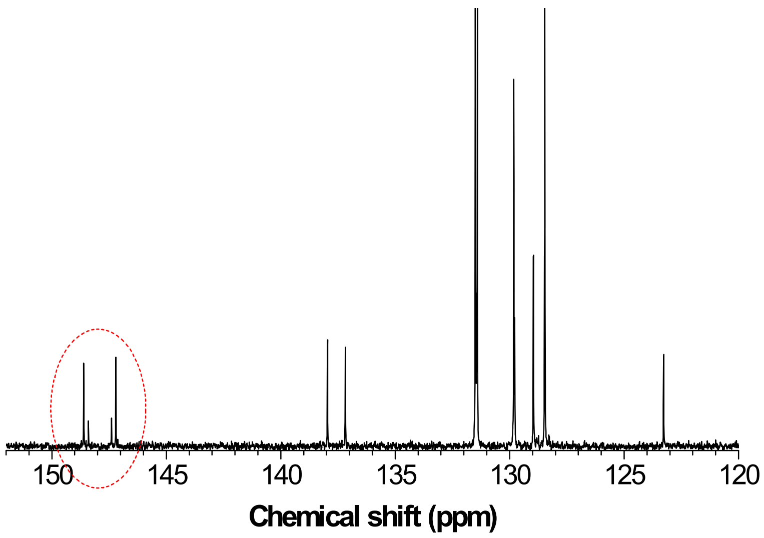

Figure S14. ^{13}C NMR spectrum of **12** in $CDCl_3$.

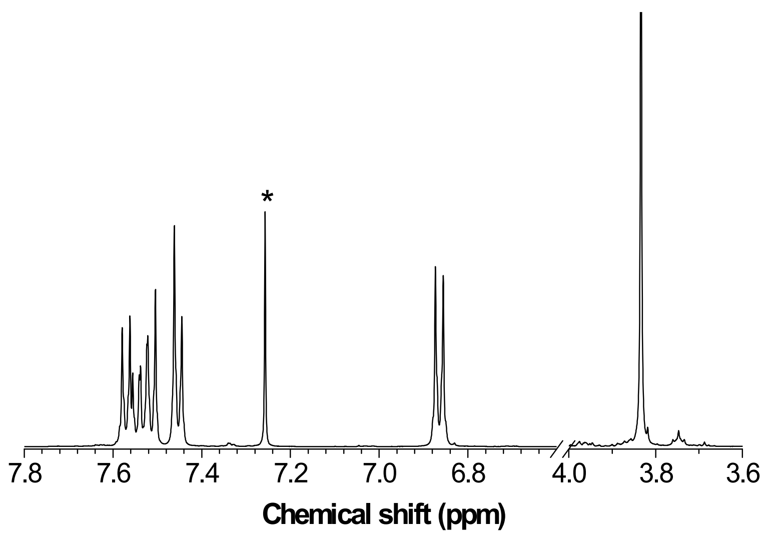

Figure S15. ^{1}H NMR spectrum of **13** in $CDCl_3$. The solvent peak is marked with asterisk.

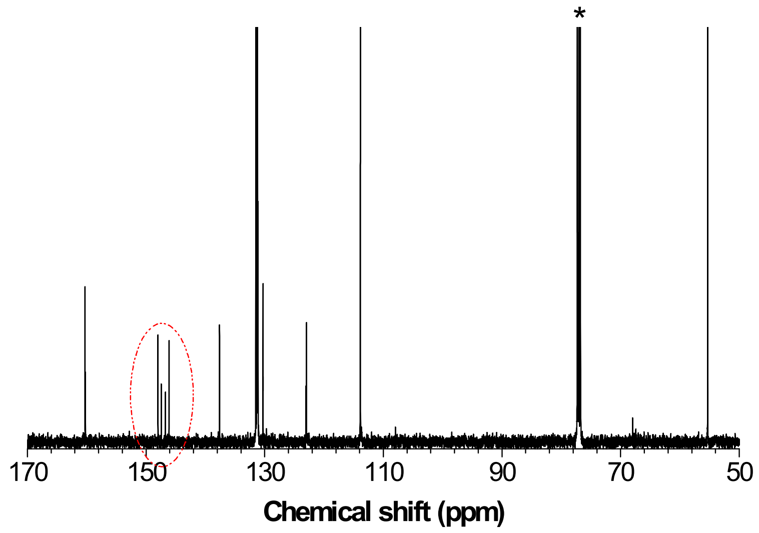

Figure S16. ^{13}C NMR spectrum of **13** in $CDCl_3$. The solvent peak is marked with asterisk.

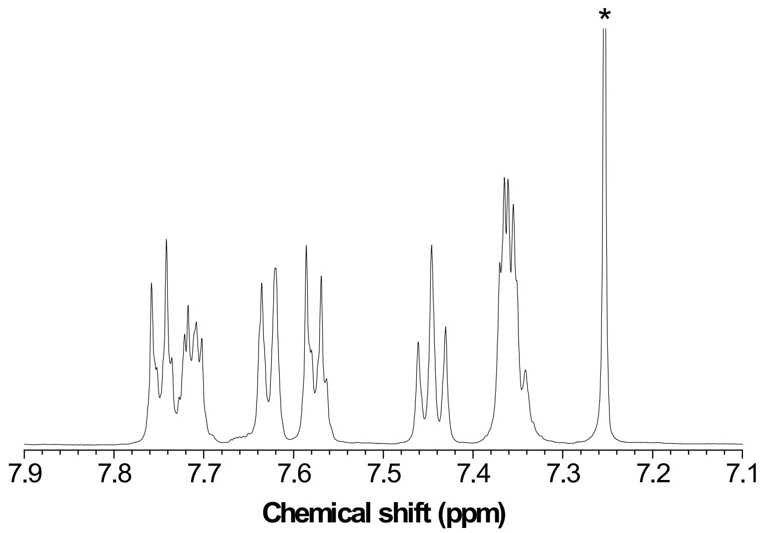

Figure S17. ^{1}H NMR spectrum of TPP-2P in $CDCl_3$. The solvent peak is marked with asterisk.

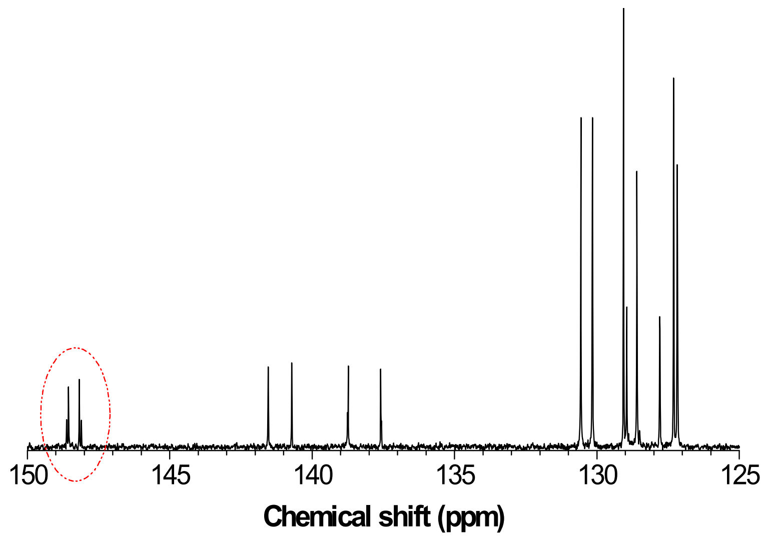

Figure S18. ^{13}C NMR spectrum of TPP-2P in $CDCl_3$.

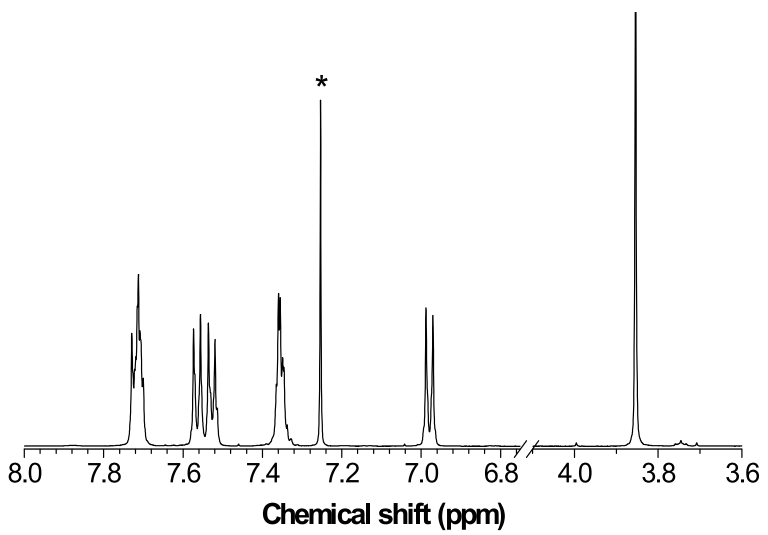

Figure S19. ¹H NMR spectrum of TPP-2PM in $CDCl_3$. The solvent peak is marked with asterisk.

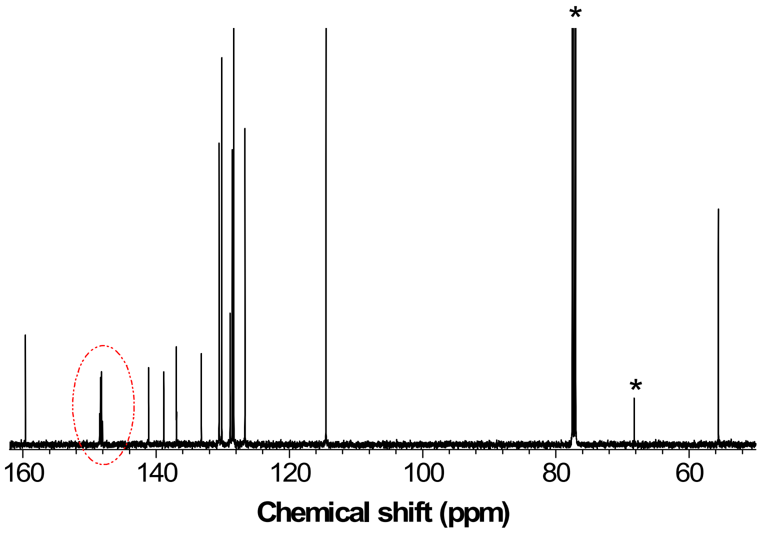

Figure S20. ¹³C NMR spectrum of TPP-2PM in $CDCl_3$. The solvent peaks are marked with asterisk.

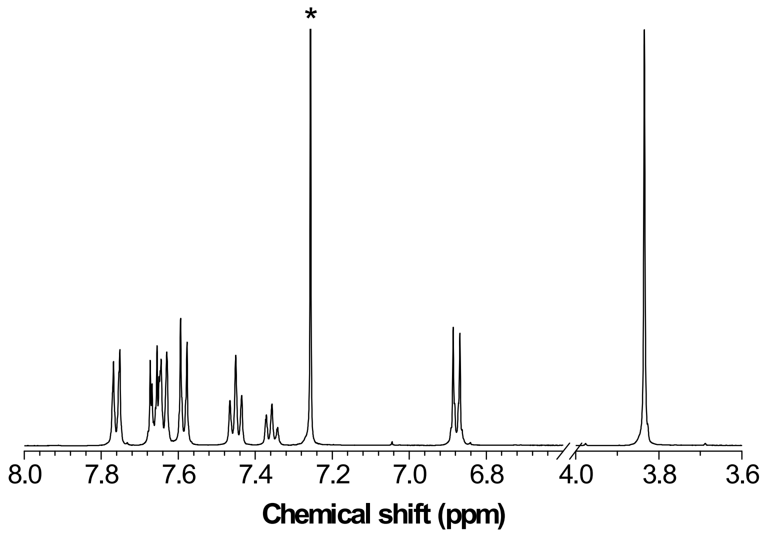

Figure S21. ^{1}H NMR spectrum of TPP-2MP in $CDCl_3$. The solvent peak is marked with asterisk.

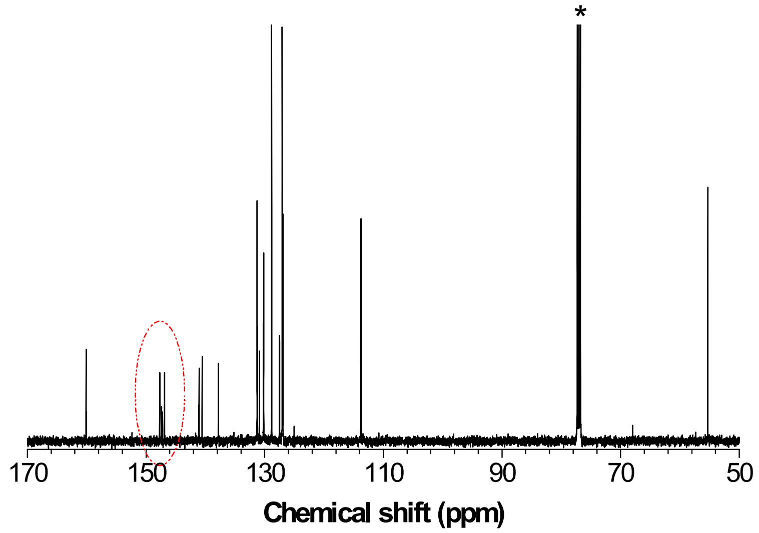

Figure S22. ^{13}C NMR spectrum of TPP-2MP in $CDCl_3$. The solvent peak is marked with asterisk.

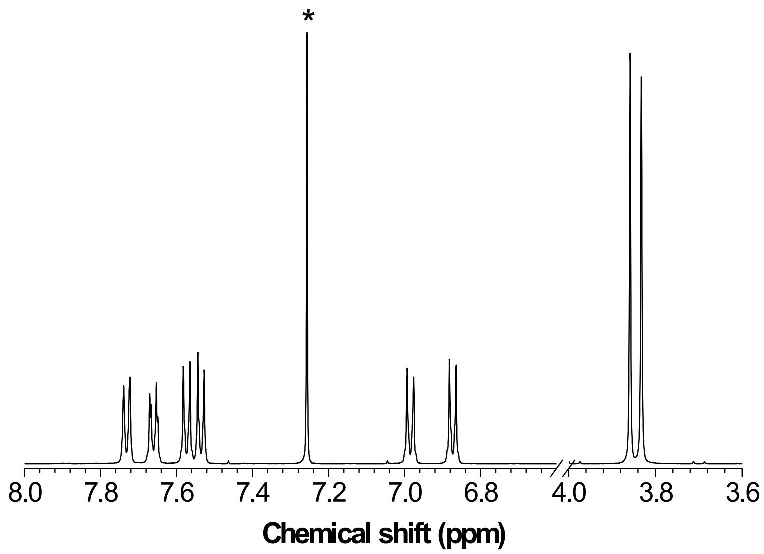

Figure S23. ^{1}H NMR spectrum of TPP-2MPM in $CDCl_3$. The solvent peak is marked with asterisk.

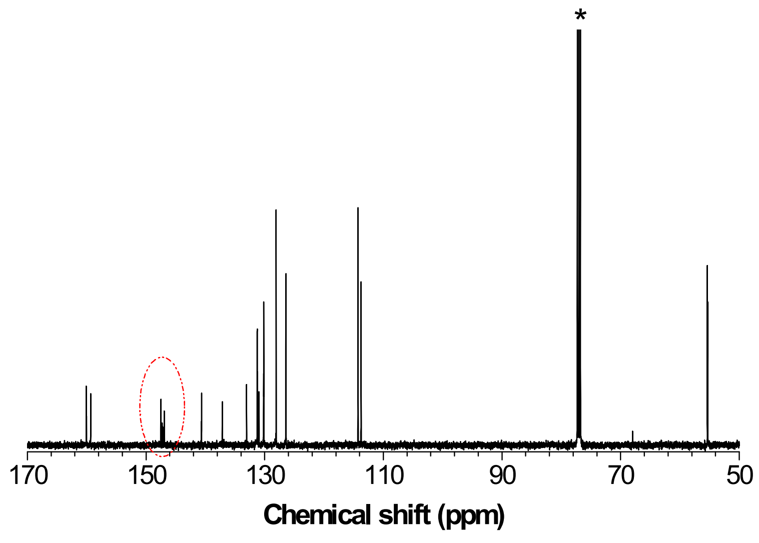

Figure S24. ^{13}C NMR spectrum of TPP-2MPM in $CDCl_3$. The solvent peak is marked with asterisk.

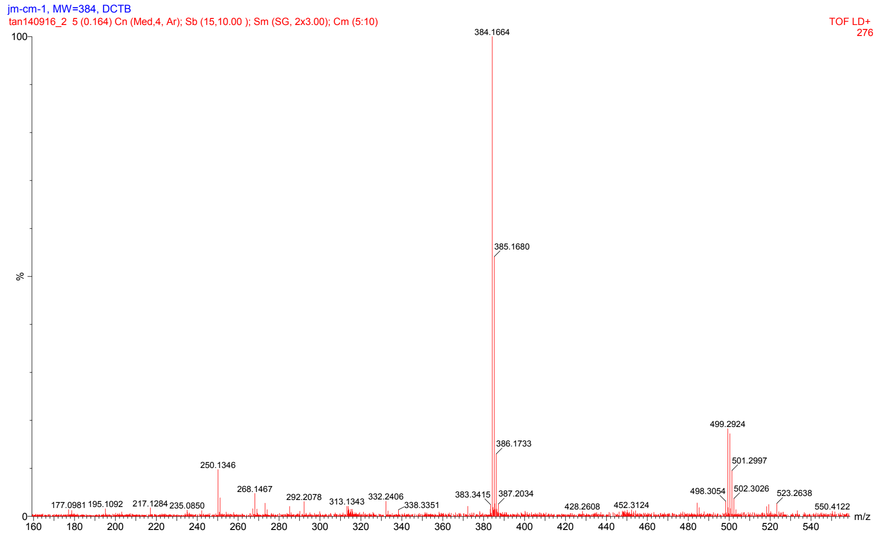

Figure S25. HRMS spectra of TPP.

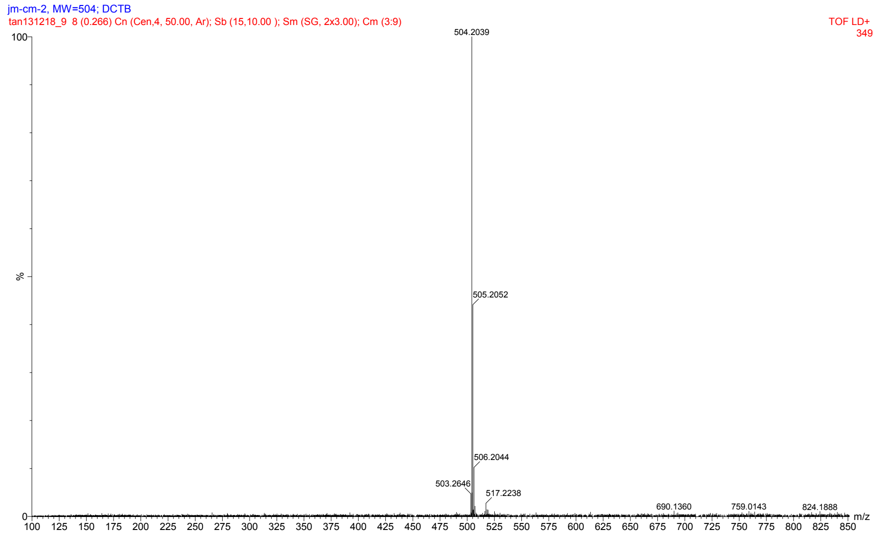

Figure S26. HRMS spectra of TPP-4M.

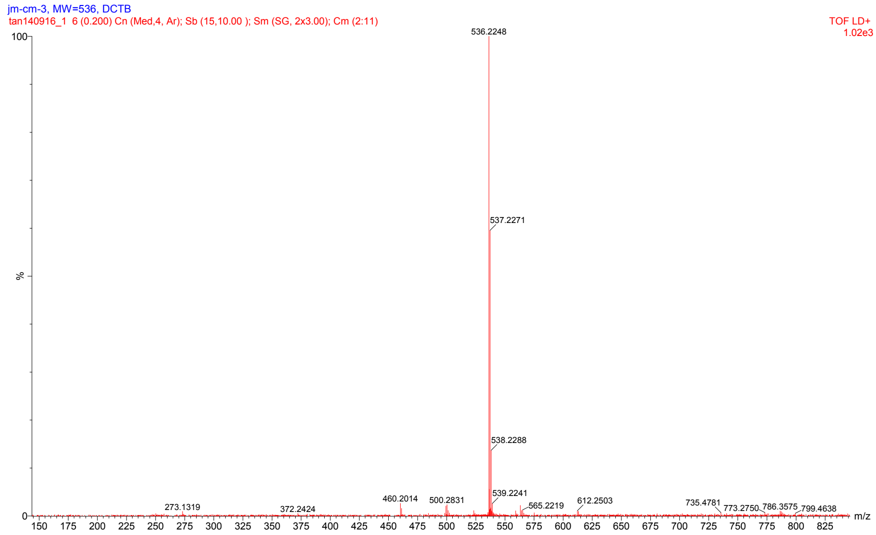

Figure S27. HRMS spectra of TPP-2P.

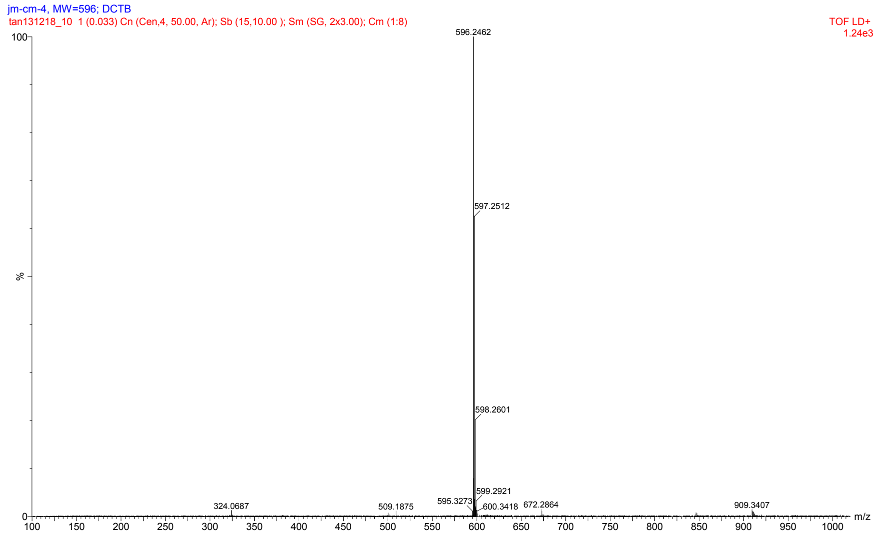

Figure S28. HRMS spectra of TPP-2PM.

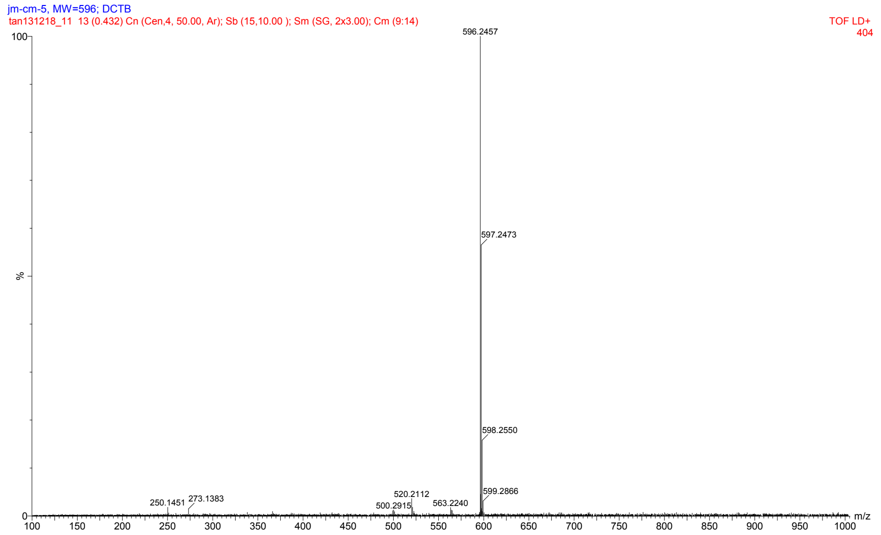

Figure S29. HRMS spectra of TPP-2MP.

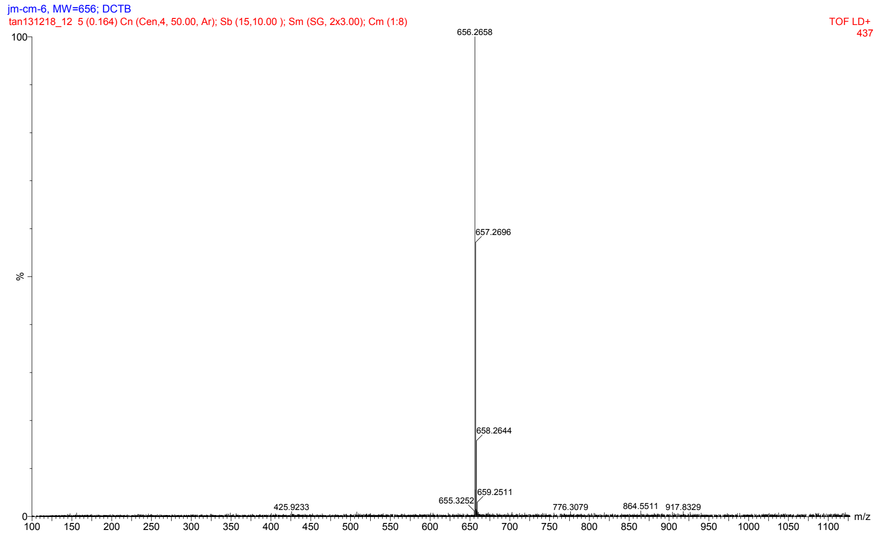

Figure S30. HRMS spectra of TPP-2MPM.

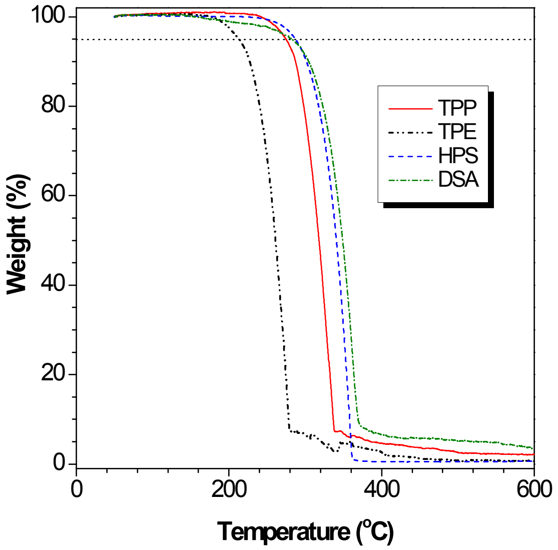

Figure S31. TGA curves of AIEgens under nitrogen at a heating rate of 10 °C/min.

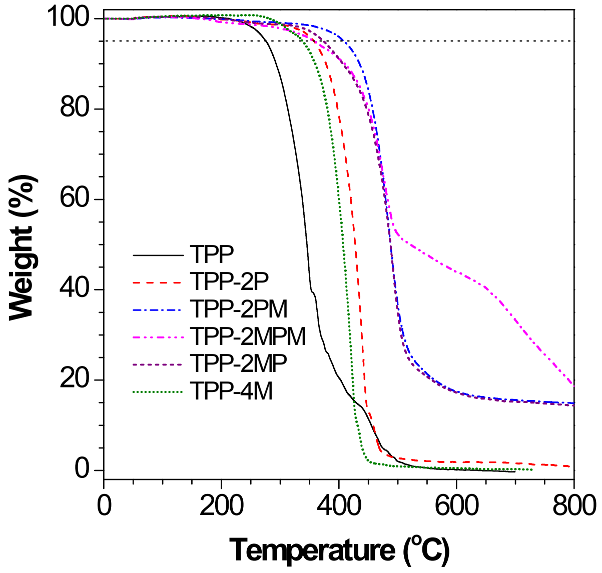

Figure S32. TGA curves of TPP and its derivatives under nitrogen at a heating rate of 10 °C/min.

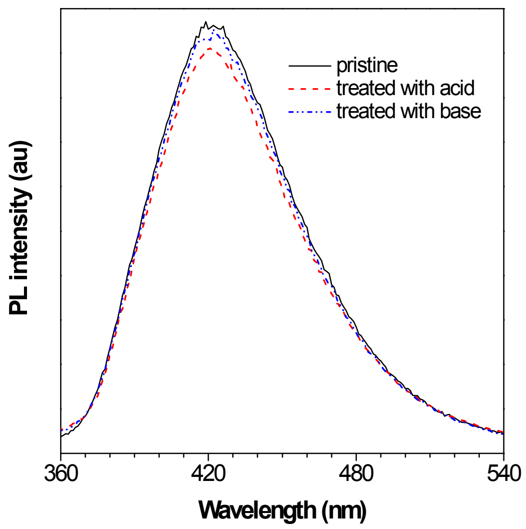

Figure S33. PL spectra of pristine and treated TPP in THF/water mixtures with ~90% water fractions. Concentration: 10^{-5} M. λ_{ex}: 338 nm. The acid and base are 0.1 mL HCl or NaOH aqueous solution (1M), respectively.

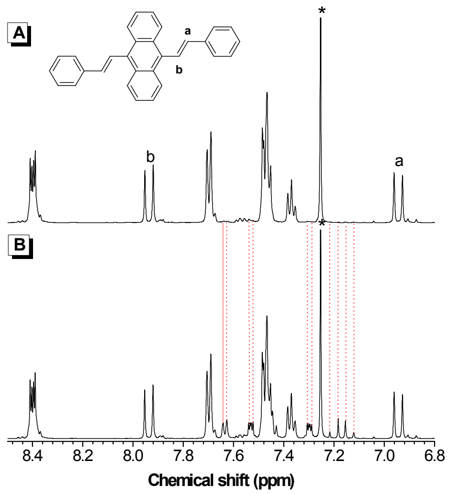

Figure S34. ^{1}H NMR spectra of DSA before (A) and after (B) irradiation by a UV light of 365 nm for 2 h in $CDCl_3$.

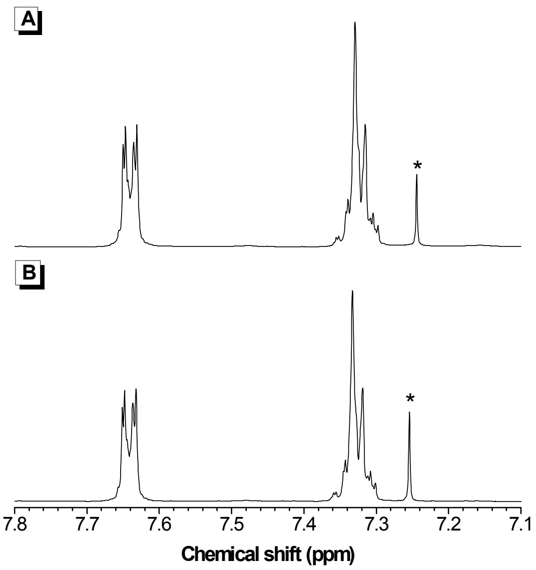

Figure S35. ^{1}H NMR spectra of TPP before (A) and after (B) irradiation by a UV light of 365 nm with a power of 1.10 mW/cm^2 for 2 h in $CDCl_3$. The solvent peaks are marked with asterisk.

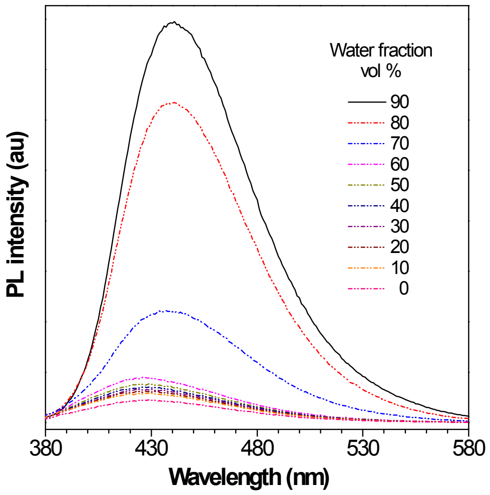

Figure S36. PL spectra of TPP-2P in THF/water mixtures with different water fraction. Concentration: 10^{-5} M, λ_{ex} = 347 nm.

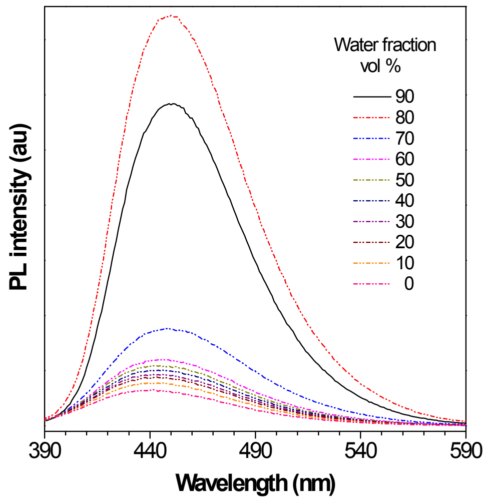

Figure S37. PL spectra of TPP-2PM in THF/water mixtures with different water fraction. Concentration: 10^{-5} M, λ_{ex} = 352 nm.

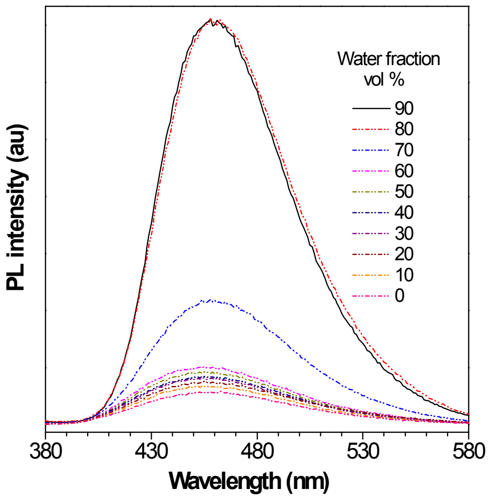

Figure S38. PL spectra of TPP-2MPM in THF/water mixtures with different water fraction. Concentration: 10^{-5} M, λ_{ex} = 362 nm.

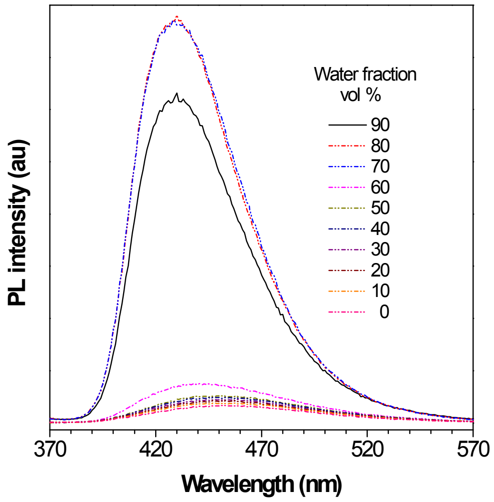

Figure S39. PL spectra of TPP-2MP in THF/water mixtures with different water fraction. Concentration: 10^{-5} M, λ_{ex} = 359 nm.

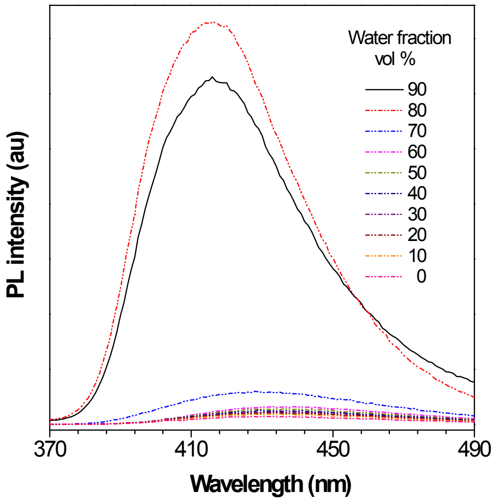

Figure S40. PL spectra of TPP-4M in THF/water mixtures with different water fraction. 10^{-5} M, λ_{ex} = 360 nm.

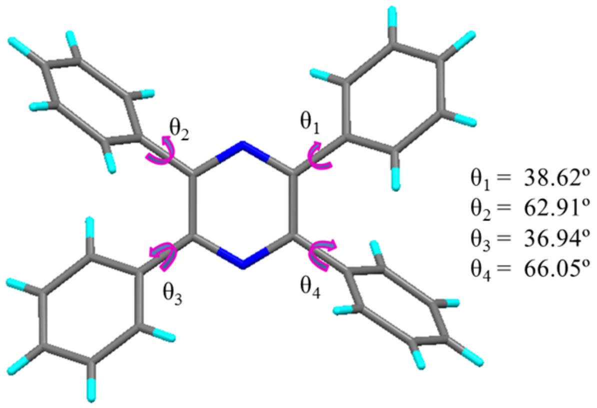

Figure S41. Molecular structures and torsion angles of TPP.

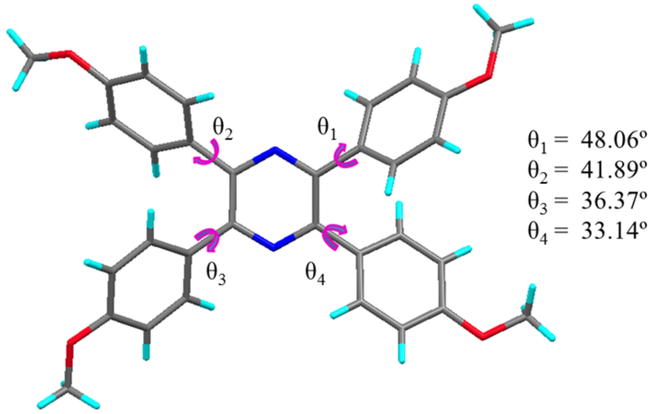

Figure S42. Molecular structures and torsion angles of TPP-4M.

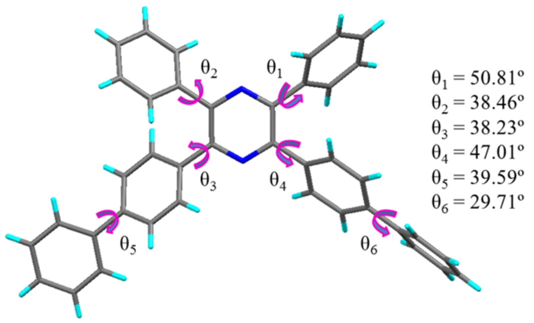

Figure S43. Molecular structures and torsion angles of TPP-2P.

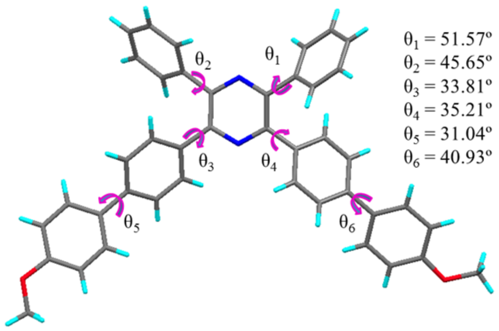

Figure S44. Molecular structures and torsion angles of TPP-2PM.

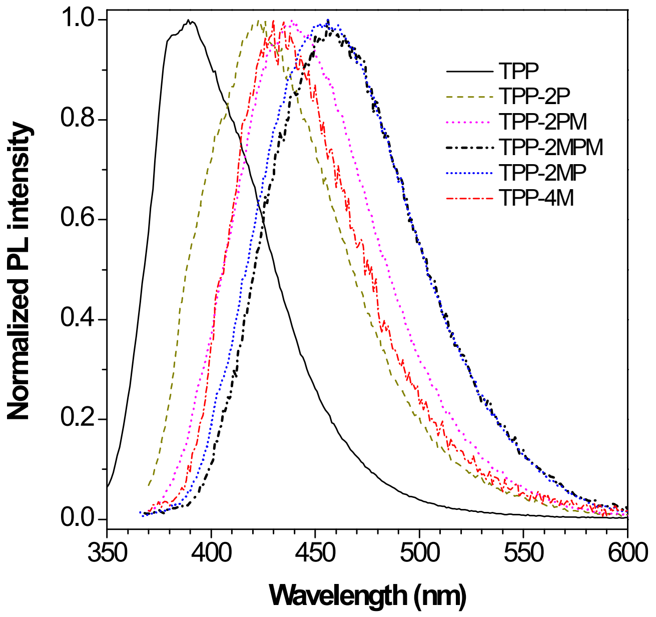

Figure S45. Normalized PL spectra of TPP derivatives in THF. Concentration: 10 μM.

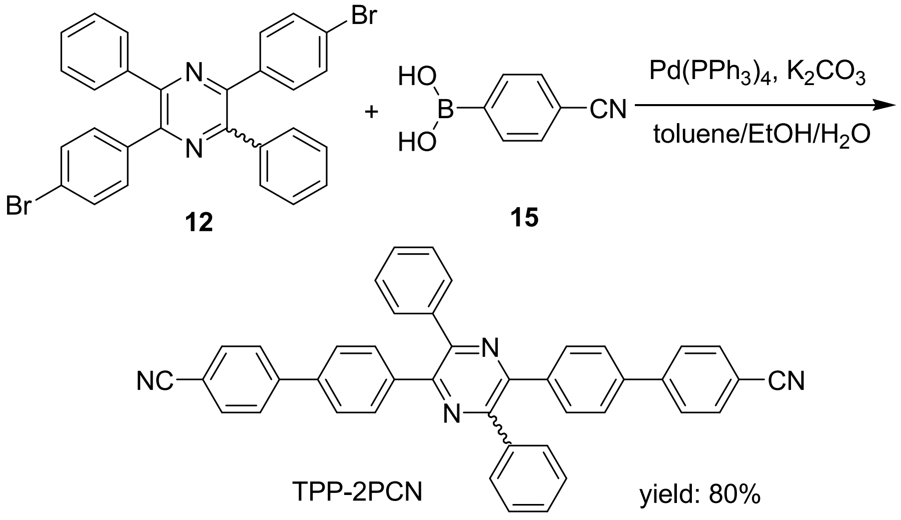

Scheme S1. Synthetic route to TPP-2PCN.

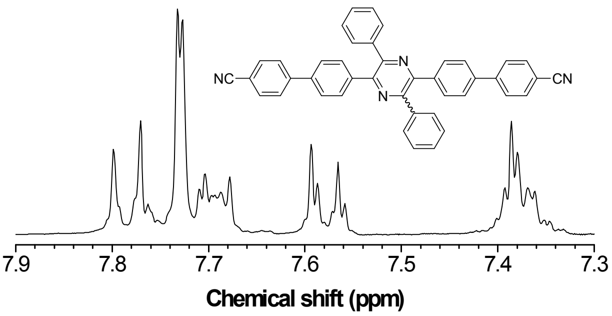

Figure S46. ^{1}H NMR spectrum of TPP-2PCN in $CDCl_3$.

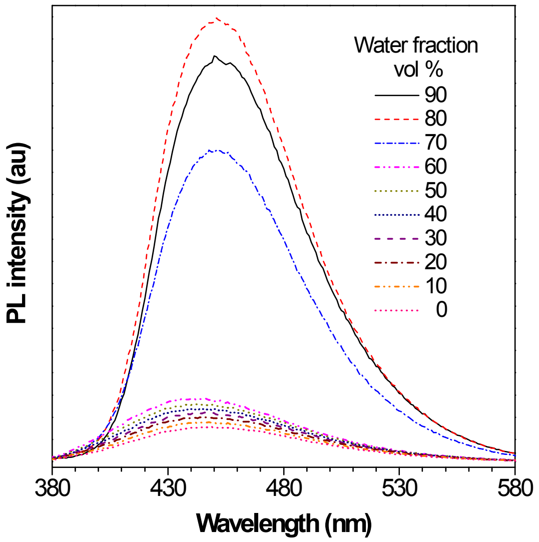

Figure S47. PL spectra of TPP-2PCN in THF/water mixtures with different water fraction. Concentration: 10^{-5} M, λ_{ex} = 347 nm.

Table S1. Optical and thermal properties of TPP and its derivatives.

	λ_{ab}/nm	$\lambda_{em,s}$/nm	$\lambda_{em,f}$/nm	$\Phi_{F,sol}$/%	$\Phi_{F,agg}$/%	Φ_F^e/%	T_d /°C
TPP	338	390	390	0.53^a	$5.70^{a,c}$	8.3	275
TPP-2P	347	423	442	1.00^b	$8.50^{b,c}$	9.2	359
TPP-2PM	352	438	453	1.20^b	$11.1^{b,d}$	18.1	410
TPP-2MPM	362	460	460	1.30^b	$16.3^{b,d}$	14.6	359
TPP-2MP	359	455	440	0.84^b	$12.5^{b,c}$	18.5	374
TPP-4M	360	433	428	0.42^a	$17.3^{a,d}$	30.7	337

[a] Measured using anthracene in ethanol as standard (Φ_F = 0.27). [b] Measured using quinine sulfate in 0.1N H_2SO_4 as standard (Φ_f = 0.54). [c] Aggregates formed in THF/water mixtures with f_w of 90%. [d] Aggregates formed in THF/water mixtures with f_w of 80%. [e] Measured using Hamamatsu Quantaurus-QY C11347 spectrometer.

Table S2. Particle sizes of TPP-based AIEgens in THF/water mixtures.[a]

f_w (%)[b]	TPP	TPP-2P	TPP-2PM	TPP-2MPM	TPP-2MP	TPP-4M
0	0	0	0	0	0	0
80	140	72	64	84	207	287
90	220	32	31	40	37	139

[a] Measured by DLS, the unit for the particle size is nm. [b] f_w = water fraction.

References

1. D. Davidson, M. Weiss and M. Jelling, *J. Org. Chem.* 1937, **2**, 328-334.
2. U. Ghosh, D. Ganessunker, V. J. Sattigeri, K. E. Carlson, D. J. Mortensen, B. S. Katzenellenbogen and J. A. Katzenellenbogen, Bioorg. Med. Chem., 2003, **11**, 629.
3. Y. Wong, K. Parthasarathy and C. Cheng, *Org. Lett.* 2010, **12**, 1736-1739.
4. Y. Xie and Z. Chen, *Synth. Commun.* 2002, **32**, 1875-1879.
